# Systematic Determinants of Global COVID-19 Burden: Longitudinal Time-Series Analysis Using Big Data-Driven Artificial Intelligence

**DOI:** 10.2196/79745

**Published:** 2025-12-29

**Authors:** Zicheng Cao, Wenjie Han, Xue Zhang, Chi Zhang, Jinfeng Zeng, Yilin Chen, Haoyu Long, Jian Chen, Xiangjun Du

**Affiliations:** 1 School of Public Health Shantou University Shantou, Guangdong China; 2 School of Public Health (Shenzhen) Shenzhen Campus of Sun Yat-sen University Shenzhen, Guangdong China; 3 School of Public Health (Shenzhen) Sun Yat-sen University Guangzhou, Guangdong China; 4 Key Laboratory of Public Health Safety, Ministry of Education School of Public Health Fudan University Shanghai China; 5 Guangdong Provincial Center for Disease Control and Prevention Guangzhou, Guangdong China; 6 Key Laboratory of Tropical Disease Control, Ministry of Education Sun Yat-sen University Guangzhou, Guangdong China; 7 Shenzhen Key Laboratory of Pathogenic Microbes and Biosafety Shenzhen Campus of Sun Yat-sen University Shenzhen, Guangdong China

**Keywords:** COVID-19, machine learning, epidemiological monitoring, vaccination coverage, environmental exposure

## Abstract

**Background:**

The COVID-19 pandemic has transitioned into an endemic phase with heterogeneous resurgences. Despite widespread vaccination and public health measures, the interplay of viral evolution, population immunity, and environmental factors drives diverse global patterns of COVID-19 burden. However, how these systematic factors dynamically shape disease transmission and severity across populations remains incompletely understood.

**Objective:**

This study aims to determine the relative contributions and temporal dynamics of viral variants, population immunity (natural infection and vaccination), environmental conditions, and public health measures in determining COVID-19 disease burden.

**Methods:**

This retrospective longitudinal time-series study used a big data-driven interpretable machine learning approach to analyze global multifaceted data across 38 countries from pandemic onset through December 31, 2022. Daily time-series data encompassing viral variants, natural infection, vaccination coverage, environmental conditions, policy interventions, health care infrastructure, and migration trends were integrated. The gradient-boosted trees (XGBoost [extreme gradient boosting]) model, coupled with Shapley Additive Explanations interpretation, quantifies the complex interdependencies and their spatiotemporal effects on 4 COVID-19 burden metrics—effective reproduction number (Rt), hospitalizations, intensive care unit (ICU) admissions, and deaths.

**Results:**

Variant-related factors dominance drives transmission/Rt (24.02%, 95% CI 10.10-66.88 contribution) but progressively attenuates across severe outcomes (4.24%, 95% CI 1.59-10.89 for ICU; 5.52%, 95% CI 1.94-15.39 for deaths). Omicron 21K and Delta 21J demonstrate exceeding baseline transmissibility by 12.2% and 3.4% respectively. Conversely, immunity-related factors show inverse patterns: natural infection contributions escalate with severity (12.82% for Rt, 14.91% for hospitalization, 21.96% for ICU [95% CI 7.36-47.55], rising to 36.00% [95% CI 10.25-78.56] for deaths). COVID-19 vaccination maintains substantial influence on severe outcomes (18.04% [95% CI 6.39-42.49] for ICU; 20.31% [95% CI 6.53-58.31] for deaths), with protective critical population thresholds: 29.9% (95% CI 29.8-29.9) coverage for transmission reduction and 72.3% (95% CI 72.2-72.8) for ICU prevention. Routine immunizations exhibit cross-protective effects, particularly the yellow fever vaccine at doses exceeding 600,000 for Rt reduction and >100,000 for ICU protection. Temperature demonstrates threshold effects: 14.95°C (95% CI 14.86-15.43) for hospitalizations and 11.89°C (95% CI 11.81-11.97) for ICU admissions. Health care infrastructure contributed 23.98% (95% CI 7.03-73.13) to hospitalization outcomes.

**Conclusions:**

The large-scale epidemiological data mining reveals previously unrecognized patterns through three innovations: (1) quantifying variant evolutionary fitness with transmission thresholds, (2) identifying dual vaccination coverage thresholds for transmission versus severe disease prevention, and (3) discovering dose-specific cross-protection from routine immunizations. Unlike black-box predictions, this interpretable framework integrates multidomain surveillance data to reveal how variants, immunity, and environment jointly shape disease burden with temporal resolution. Real-world applications include tiered vaccination strategies targeting specific coverage goals, variant surveillance prioritizing lineages with demonstrated fitness in contemporary immunity contexts, and expanding routine immunization programs as pandemic preparedness measures. This framework provides quantifiable benchmarks for adaptive pandemic response across immunization strategies, variant surveillance, and health care capacity planning.

## Introduction

The COVID-19 pandemic, instigated by the SARS-CoV-2 virus, has precipitated an unprecedented global health crisis, fundamentally altering societal behaviors and interactions worldwide [1-3]. Approaching its fifth year, SARS-CoV-2 continues global evolution, manifesting heterogeneous resurgence patterns that have established COVID-19 as an endemic challenge. Sustained surveillance and adaptive public health strategies are needed to address the multiple determinants of disease transmission and outcomes. The global outbreak has demonstrated significant temporal variability, driven by multiple interacting factors that contribute to diverse disease outcomes, collectively referred to as COVID-19 burden metrics [4,5]. The emergence of immune-evasive variants, coupled with waning immunity from vaccination and prior infections, has created a dynamic epidemiological landscape where COVID-19 burden is assessed through multiple indicators, including the effective reproduction number (Rt), hospitalizations, intensive care unit (ICU) admissions, and mortality rates [6,7]. These metrics display marked heterogeneity across populations, reflecting the pandemic’s complexity.

Existing literature has extensively explored the effects of nonpharmaceutical interventions (NPIs), vaccination strategies, population mobility, and the strategic allocation of health care resources in curtailing the pandemic’s impact [8-14]. However, despite extensive vaccine coverage and the development of variant-specific boosters, heterogeneous COVID-19 burden persists, challenging our understanding of population-level immunity dynamics [15]. The complexity arises from multiple interacting factors —hereafter termed “systematic factors”, defined as the interconnected viral, host, environmental, and intervention-related determinants that collectively shape disease transmission and severity patterns [16-19]. While vaccination remains essential for controlling variant spread and reducing severe outcomes, its protective efficacy demonstrates temporal decline and variant-specific limitations [20-22]. The immunological landscape is further diversified by heterogeneous immunity patterns across populations, shaped by natural infection history, vaccination coverage, and potential cross-protection from routine immunizations [23-28]. Environmental factors, particularly temperature and humidity variations, also modulate transmission dynamics [29-31].

Recent artificial intelligence (AI) advances have enabled integration of high-dimensional pandemic data for mechanistic understanding [32-34]. Explainable AI XAI) methods, particularly those applied in clinical decision support systems, have demonstrated the capacity to balance predictive accuracy with interpretability—a critical requirement for translating algorithmic insights into actionable clinical and public health decisions [35,36]. Machine learning models, particularly ensemble methods, demonstrate superior performance in capturing nonlinear interactions between viral evolution, population immunity, and environmental factors [37,38]. However, a critical limitation persists: many high-performing models function as “black boxes,” obscuring mechanisms and limiting actionable public health insights [39,40]. This has motivated a paradigm shift toward interpretable machine learning, where Shapley Additive Explanations (SHAP) enables mechanistic decomposition of complex predictions into individual feature contributions [41,42]. Recent studies have applied SHAP-interpreted ensemble models to disentangle vaccination, variant, and policy effects on COVID-19 outcomes, demonstrating that interpretable approaches can simultaneously achieve high performance while revealing factor-specific effects and threshold dynamics [43-45].

Current research predominantly focuses on discrete disease outcomes or specific contributing factors, often using cross-sectional or short-term study designs that inadequately capture the pandemic’s temporal dynamics. These fragmented approaches are particularly problematic in the context of co-circulating variants and heterogeneous immune landscapes, which interact with environmental conditions in complex, time-varying patterns. Three key questions remain inadequately addressed: (1) “How do relative contributions of systematic factors shift across disease severity outcomes?”, (2) “What quantitative thresholds exist where vaccination coverage, natural infection rates, and environmental parameters transition from promoting to suppressing disease burden?”, and (3) “Can cross-protective effects from routine immunizations be systematically quantified with dose-response relationships?” Addressing these gaps requires integrated longitudinal analysis of multifactorial influences on COVID-19 burden metrics.

To address these questions, this study uses big data analytics and interpretable machine learning, analyzing longitudinal datasets from 38 nations to evaluate multifactorial influences on COVID-19 burden metrics. Using extreme gradient boosting XGBoost (extreme gradient boosting) regression models integrated with SHAP [46-48], we systematically evaluate variables, including policy interventions, migratory trends, health care infrastructure, environmental parameters, viral variant characteristics, vaccination campaigns, and non-COVID-19 immunization programs. We selected XGBoost+SHAP because: (1) XGBoost captures complex nonlinear interactions without requiring prior functional form specification—critical for unknown variant-immunity-environment relationships; (2) SHAP yields consistent local/global attributions that can be aggregated over time and domains to derive interpretable thresholds and uncertainties; (3) our objective prioritizes mechanistic interpretation (quantifying factor contributions, identifying thresholds) over predictive optimization, making interpretable ensemble learning optimal. This approach disentangles the direct and indirect effects exerted by multifaceted determinants on COVID-19 burden metrics. Our methodological approach harnesses large-scale, time-series big data, facilitating a comprehensive examination of multifactorial influences on COVID-19 severity over time, while integrating multiple data sources that capture the evolving interplay between pathogen characteristics, host immunity, and environmental conditions. We hypothesize that systematic COVID-19 burden determinants exhibit hierarchical, threshold-dependent effects that shift across disease severity outcomes, with viral factors dominating transmission while immunity-related and environmental factors increasingly modulate severe disease through nonlinear mechanisms. Through multidimensional big data-driven interpretable machine learning integrating viral surveillance, immunization records, and environmental monitoring, we aim to quantify the dynamic influences of these factors on evolving disease burden for evidence-based pandemic preparedness.

## Methods

### Study Design

This study uses a retrospective longitudinal, multicountry observational design using big data analytics and interpretable machine learning to systematically evaluate the multifactorial determinants of COVID-19 burden metrics. The analytical framework integrates heterogeneous time-series data from 38 countries across 5 continents, spanning from the onset of confirmed cases in each country through December 31, 2022. Our approach comprises four sequential phases: (1) multisource data collection and harmonization, (2) temporal feature engineering with lag optimization, (3) XGBoost-based modeling with time-series cross-validation, and (4) SHAP-based interpretability analysis to quantify dynamic temporal influences and identify threshold effects. The study leverages publicly available, deidentified aggregate-level epidemiological data, thereby circumventing individual participant recruitment. This design enables a comprehensive examination of the dynamic relationships between viral evolution, population immunity, environmental conditions, and disease outcomes from a global perspective. Reporting was guided by the Critical Appraisal Skills Programme checklist for descriptive studies; the completed checklist is provided in the Multimedia Appendix 1.

### Data

#### Setting and Study Period

The study encompasses 38 countries distributed across 5 continents (detailed in Table S1 in Multimedia Appendix 2), selected based on the highest regional disease burden as measured by cumulative cases, hospitalizations, and mortality rates within their respective geographical areas. Collectively, these 38 countries represented over 60% of both the global population and the global COVID-19 burden as of December 31, 2022, thereby providing substantial representation of global pandemic patterns despite potential surveillance heterogeneity across nations. The observation period extends from the date of the first confirmed COVID-19 case in each country. This endpoint represents a methodologically justified cutoff, corresponding to the final period of consistent global surveillance prior to the World Health Organization’s (WHO’s) recategorization of COVID-19’s public health emergency status in early 2023. Post-2023, substantial heterogeneity in national reporting practices emerged, with many countries discontinuing systematic documentation of key burden metrics, thereby compromising data completeness and cross-national comparability. The study period encompasses multiple global pandemic waves, including the emergence and circulation of major variants of concern (Delta and Omicron lineages), providing sufficient temporal depth to capture recurring patterns in transmission dynamics and disease severity across diverse epidemiological contexts.

#### Data Collection and Scope

This research compiles a comprehensive dataset encompassing key indicators of COVID-19 burden metrics across 38 countries. These indicators include Rt, hospitalizations, ICU admissions, and deaths, tracking the pandemic’s trajectory from the onset of confirmed cases in each country through December 31, 2022.

#### Outcome Variables and Assessments

This study defines four primary outcome variables representing the spectrum of COVID-19 disease burden, assessed as daily time-series data for each country: (1) Transmission potential (effective reproduction number [Rt], representing the average number of secondary infections generated by each infected individual at time *t*); (2) Hospitalization burden (daily new hospital admissions per million population attributable to COVID-19); (3) Intensive care burden (daily new ICU admissions per million population for COVID-19 patients); and (4) Mortality burden (daily COVID-19-attributed deaths per million population).

#### Data Categories and Sources

#### Overview

Previous studies have established various factors that may influence COVID-19 disease burden, including viral variants, vaccination coverage, population mobility, NPI policies, and health care capacity [8-14]. Additionally, some factors such as routine immunization history and environmental conditions have been hypothesized to affect COVID-19 outcomes, though their impacts remain to be fully elucidated [27-30]. To comprehensively examine these established and potential determinants, the study systematically collects data from diverse, reliable platforms, encompassing several epidemiological aspects related to COVID-19 burden metrics. The analysis includes 65 independent potential factors serving as covariates in the modeling framework. Specifically, the collected data encompasses the following sections.

#### COVID-19 Burden Data

Daily data on Rt, hospitalizations*,* ICU admissions, and mortality rates were sourced from Our World in Data via CSV download with daily extraction timestamps. Data validation involved cross-checking against Johns Hopkins University CSSE COVID-19 Data Repository and national health ministry reports for 10 randomly selected country-date combinations monthly, with >95% concordance observed.

#### Natural Infection–Related Group

Two epidemiological factors are extracted from Our World in Data: Daily new cases per million population (measured as 7-day rolling average to smooth reporting irregularities) and cumulative cases per million population (calculated as a cumulative sum from pandemic onset, serving as a proxy for population-level exposure and acquired immunity).

#### Variant-Related Group

We monitor the temporal composition of 13 dominant strains during the pandemic, focusing on variants that demonstrate cross-continental transmission capabilities and are classified as variants of concerns by the WHO. Specifically, for each of the 38 countries, we calculate the weekly proportions of major SARS-CoV-2 lineages, including Delta variants (Nextstrain: 21A [B.1.617.2], 21I [AY.4], 21J [AY.2]) and Omicron variants (Nextstrain: 21K [BA.1], 21L [BA.2], 21M [BA.3], 22A [BA.4], 22B [BA.5], 22C [BA.2.12.1], 22D [BA.2.75], 22E [BQ.1], 22F [XBB], and 23A [BA.2.86]). The proportion is calculated by dividing the number of sequences for each variant by the total number of sequences submitted that week within each country, using data sourced from covariants via weekly automated data pulls. Weekly proportions are forward-filled to daily resolution to align with the outcome variable’s temporal granularity. Sequencing coverage varies by country, with variant proportions assumed representative of circulating strains based on established genomic surveillance methodologies. These lineages are selected based on the Nextstrain naming system, which provides sequential identifiers reflecting the temporal emergence of each lineage, with corresponding Pango lineage designations provided in brackets for clear reference.

#### COVID-19 Vaccine–Related Group

Six vaccination-related factors are obtained from Our World in Data, including daily vaccine doses administered (total doses given on each day), cumulative vaccine doses (running total since vaccination campaign initiation), rates of at least one dose (percentage of population with ≥1 vaccine dose), complete vaccination coverage (percentage meeting initial full vaccination criteria), and booster dose administration (percentage receiving additional doses beyond primary series). All metrics are expressed as percentages of the total national population or absolute counts per million, sourced from national immunization registries via Our World in Data aggregation.

#### Non–COVID Vaccine–Related Group

Ten non-COVID vaccine indicators are included, representing annual doses of vaccines administered through national immunization programs. These include Bacillus Calmette–Guérin (BCG), Diphtheria tetanus toxoid and pertussis, Hepatitis B, Haemophilus influenzae type b, human papillomavirus, Measles (measles-containing vaccine), Pneumococcal (pneumococcal conjugate vaccine), Poliomyelitis, Rubella (rubella-containing vaccine), Rotavirus, and yellow fever vaccines (YFVs), which are sourced from the WHO’s vaccine coverage reports. Data represent total annual doses administered nationally, reported by member states through WHO-UNICEF (United Nations Children's Fund) joint reporting forms. Annual values are uniformly distributed across days within each calendar year for temporal alignment with daily COVID-19 metrics.

#### Policy-Related Group

Sixteen NPIs are tracked daily using the Oxford COVID-19 Government Response Tracker. Each intervention is quantified on a (0,100) index scale to reflect the stringency of implemented measures (with 0 indicating no policy and 100 indicating maximum stringency, calculated via ordinal coding of policy intensity and geographical scope as detailed in Table S2 in Multimedia Appendix 2). Daily index values are extracted via API access with version control timestamps. Further details on these NPIs and the methodology for calculating the stringency index are provided in Multimedia Appendix 2.

#### Health Care–Related Group

Nine health care–related factors are collected, including annual population numbers (total population), density (persons per km^2^), gross domestic product (GDP) per capita (constant US $), percentage of population aged 65 years and older, health care expenditure (percentage of GDP), numbers of doctors, nurses, pharmacists (per 1000 population), and hospital beds (per 1000 population) are collected from Our World in Data and World Bank Open Data. Annual values are carried forward as constant daily values within each calendar year, assuming stable health care infrastructure characteristics over short-term pandemic periods.

#### Environmental Group

Five environmental factors are analyzed, including daily maximum, minimum, and average temperatures (°C), relative (%), and absolute humidity (g m^–3^), obtained from the National Oceanic and Atmospheric Administration Global Surface Summary of the Day dataset. For each country, meteorological data are extracted from the city’s primary weather station, serving as a representative measure of national-level environmental conditions. Missing daily values (<3% of observations) are imputed using linear interpolation between adjacent days.

#### Migration-Related Group

Four migration-related factors are analyzed, including national-level monthly data on domestic air passenger traffic (total passengers on domestic flights) and international air passenger traffic (total passengers on international inbound/outbound flights). These data are obtained from the Official Aviation Guide via licensed data subscription. Monthly passenger counts are distributed uniformly across days within each month for temporal alignment.

#### Data Preprocessing and Harmonization

To ensure consistency, all time scales standardize to daily intervals using pandas time series methods, facilitating seamless integration and analysis of diverse datasets. Prior to analysis, all collected data undergo a thorough preprocessing procedure, which includes the following: (1) Missing data handling: For outcome variables (Rt, hospitalizations, ICU admissions, deaths), days with missing values are excluded from country-specific analyses (affecting <5% of total country-day observations). For predictor variables, missing values are imputed using carry-forward imputation for time-invariant factors (health care infrastructure) and linear interpolation for time-varying factors (environmental conditions, mobility). (2) Outlier detection and correction: Values exceeding 5 SDs from the country-specific rolling 30-day mean are flagged for manual verification against source databases. Confirmed data entry errors are corrected via source re-extraction; legitimate extreme values (eg, genuine outbreak peaks) are retained. (3) Temporal alignment: All variables are synchronized to daily resolution using forward-filling (policy indices, health care factors), uniform distribution (monthly migration data, annual vaccination data), or linear interpolation (environmental data gaps) as appropriate to variable characteristics.

#### Study Size Determination

The study size is determined by the temporal scope of the pandemic observation period and data availability across selected countries. The final analytical dataset comprises 38,908 country-day observations (38 countries × mean 1023 days per country, range: 999-1074 days depending on pandemic onset date). This sample size provides sufficient statistical power (>99% power to detect effect sizes of Cohen *f*^2^ ≥ 0.01 at *α*=0.05) for modeling nonlinear relationships between 65 predictor variables and 4 outcome variables across diverse epidemiological contexts. The multicountry longitudinal design enables both cross-sectional (between-country) and temporal (within-country) variation exploitation, enhancing generalizability and robustness of identified patterns.

#### Lag Period Optimization

The analysis systematically quantifies the lag effects of key predictors on COVID-19 burden metrics, accounting for the inherent temporal dynamics of epidemiological factors. The analytical approach differentiates between incremental and cumulative measures based on their distinct temporal patterns, necessitating tailored lag optimization methods: For incremental measures (daily new cases, daily vaccination doses, complete vaccination rates, and booster administration), which exhibit stochastic fluctuations and potential autocorrelation decay, partial autocorrelation function analysis identifies optimal lag periods by isolating direct temporal dependencies while controlling for intermediate lags—appropriate for variables where recent values may influence outcomes through short-memory processes. This method captures the strongest temporal correlations while controlling for intermediate lag effects, providing precise estimates of the most influential temporal windows for each factor (detailed in Figure S1 in Multimedia Appendix 2). For cumulative infection counts, which represent monotonically increasing exposures with long-memory characteristics (population immunity accumulation), a lag-specific regression framework determines the optimal lag structure for each COVID-19 burden metric through systematic Akaike Information Criterion comparison—appropriate for variables where the relevant temporal window (eg, duration of immune protection from cumulative exposure) may vary by outcome severity and requires outcome-specific optimization. This approach systematically evaluates model fit across the lag space using the Akaike Information Criterion, enabling the identification of the most parsimonious lag period that maximizes the explanatory power for each burden outcome. Figure S2 in Multimedia Appendix 2 illustrates the comprehensive lag optimization process. For migration-related factors, specifically domestic and international air passenger traffic available at monthly temporal resolution, the analysis incorporates both current-month and one-month lagged features, as the delayed impact of population movement typically manifests within this timeframe [49].

The identified optimal lag periods integrate into the temporal feature set, ensuring capture of the most relevant temporal associations between predictors and outcomes. This unified and systematically organized dataset serves as the foundation for a comprehensive analysis of the multidimensional factors underlying global COVID-19 burden metrics.

### Modeling for Explaining COVID-19 Burden Metric

#### Overview of the Machine Learning Framework

This study uses a multivariate time-series machine learning framework to develop an explanatory model that analyzes the relationships between COVID-19 burden metrics and their determinants across 38 countries. This framework enables efficient processing and integration of large-scale heterogeneous data, revealing previously unidentified epidemiological patterns and correlations. The interpretable machine learning approach is well-suited for quantifying the individual effects of multidimensional factors on COVID-19 outcomes. This methodology enhances our understanding of the underlying mechanisms driving pandemic dynamics while demonstrating the effectiveness of interpretable machine learning in analyzing complex epidemiological relationships.

#### Model Construction and Variables

For each country, daily time-series data of Rt, hospitalizations, ICU admissions, and deaths serve as target variables to build the XGBoost regression model. The analysis generates a comprehensive feature set that delineates the relationships between factors and outcomes across all countries. The model integrates temporally-aligned data across 8 domains: natural infection-related (2 factors), variant-related (13 factors), COVID-19 vaccination (6 factors), non–COVID-19 vaccination factors (10 factors), policy-related (16 factors), health care–related (9 factors), environmental (5 factors), and migration-related factors (4 factors) (as detailed in the Data section). All 65 factors serve as covariates in the regression framework, with no derived or composite variables constructed beyond the lag-transformed features described in the lag optimization section. The modeling approach treats these heterogeneous time-series factors as temporally-aligned covariates, enabling XGBoost to capture complex nonlinear interactions and temporal dependencies in their relationships with the 4 outcome variables. This multidomain integration enables a holistic analysis of pandemic dynamics [50].

#### XGBoost Model Training and Cross-Validation

The XGBoost model serves as the primary analytical tool in this study [48], selected for its robust fitting capabilities and widespread application in epidemiological research [50]. This model excels in handling complex, multidimensional time-series data, making it particularly suitable for this application. The model is implemented using the XGBoost Python library (version 1.7.3) in a Python 3.11 environment with scikit-learn (version 1.7.2) for cross-validation and hyperparameter tuning. To ensure robust model performance and generalizability, we use a nested time-series cross-validation approach accounting for both temporal dependencies and cross-national heterogeneity: (1) Country-stratified temporal splitting: For each country, the chronologically ordered daily observations are partitioned into 5 consecutive folds, preserving temporal sequence to avoid data leakage. This yields a rolling-window validation scheme where each fold serves sequentially as the validation set while prior folds constitute the training set. (2) Cross-national validation: Models trained on data from all countries are validated using the held-out temporal fold from each country, enabling assessment of generalization across both time and geographical contexts. (3) Performance aggregation: Final performance metrics (mean absolute error, root-mean-square error, and *R*^2^) are calculated as the mean across all validation folds and countries, with 95% CIs derived via bootstrapping (1000 resamples).

### Factor Analysis and Model Interpretation

#### Overview

To quantify key factors influencing COVID-19 burden metrics and decode their relationships, we integrate SHAP into our machine learning framework [51]. SHAP, an interpretable machine learning technique, facilitates a detailed examination of how multidimensional variables interact with and influence outcome variables within the XGBoost model. SHAP quantifies the contribution of each factor to the outcome variable. This approach provides a granular view of the dynamic interplay among various factors over time, enabling the dissection of the relationships that define COVID-19 burden metrics. Through this methodology, SHAP offers a quantitative measure of factor contributions and decodes complex interactions among multiple variables, thus enhancing our understanding of the factors influencing COVID-19 outcomes.

#### SHAP Value Computation

The SHAP method, inspired by game theory, explains the predictions of machine learning models by assigning a value to each input feature [46,52]. This value, known as the SHAP value, signifies how each feature contributes to the prediction of a specific data point [53]. SHAP values are computed using the TreeExplainer algorithm (SHAP Python library version 0.42.1), which leverages the tree structure of XGBoost models to efficiently calculate exact Shapley values. It identifies factors with positive influence (promotive effect, SHAP values > 0) and those with negative influence (inhibitory effect, SHAP values < 0) on the predicted outcome variable. The computation of SHAP values is formalized as follows:

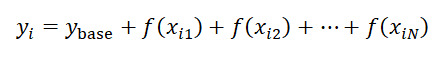



where the *i-*th sample of the *N*-th feature is defined as *x_iN_*, the reference value of the model (the mean value of the sample target variable) is *y*_base_, and *f*(*x_iN_*) is the SHAP value of *x_iN_*. We compute SHAP values for each influencing factor throughout the entire time series for every country. These values indicate the magnitude and direction of each feature’s effect on the model output (fitted values of COVID-19 burden metrics).

#### Factor Importance Quantification: SHAP-PCA Integration

The overall importance of factors in this study is quantified by averaging the absolute SHAP values across all time points for each country. These factors are categorized into 8 distinct groups, including policy-related, migration-related, and COVID-19 vaccine–related factors. Principal component analysis (PCA), a dimensionality reduction technique suitable for high-dimensional data, reduces the SHAP value dimensions within each category to a singular, representative impact value. This process effectively transforms the multifaceted nature of these variables into a single dimension, reflecting their cumulative effect—a composite impact indicator of the diverse factors. The Mann-Whitney *U* test, a nonparametric method, is used to assess the statistical significance of differences between factor categories. This test is selected for its robustness against nonnormal distributions and its capability to handle independent samples of different sizes. Pairwise comparisons are conducted between all factor categories, with statistical significance defined as *P*<.05. This rigorous statistical approach enables quantitative evaluation of the relative importance of different factor categories in determining COVID-19 burden metrics.

#### Temporal Dynamics and Global Trends

The analysis plots simplified category-wise SHAP values across the time series to assess temporal dynamics of factor categories, providing a visual representation of category heterogeneity over time. Within-category Z-score normalization of SHAP values, constrained to a (–1,1) range, enhances the visualization of temporal patterns and facilitates meaningful intercategory comparisons. This standardization procedure effectively highlights the relative temporal variations within each factor category while maintaining their interpretability and comparative significance. This approach aids in evaluating fluctuating category dependencies on various COVID-19 burden metrics. Global trends emerge through the analysis of average PCA-reduced SHAP values across all countries.

#### SHAP Dependency Analysis and Effect Curves

SHAP partial dependence plots reveal dependency relationships between high-contribution factors and COVID-19 burden metrics (Multimedia Appendix 3) [53]. These plots demonstrate the marginal relationships between individual factors and COVID-19 burden metrics. Generalized additive models (GAM) with cubic spline smoothing are fitted to the SHAP values across the feature range to quantify these relationships systematically. GAM fitting is implemented using the pyGAM Python library (version 0.8.0) with 20 spline basis functions and a second-order penalty (*λ* optimized via generalized cross-validation). The GAM-fitted effect curves, accompanied by 95% CIs, capture nonlinear patterns in the feature-outcome relationships. The 95% CIs are derived via simultaneous inference procedures accounting for multiple comparisons across the feature range, providing conservative uncertainty bounds for threshold identification. These curves emphasize trend transitions across the SHAP=0 decision boundary, which represents the critical threshold between risk reduction (SHAP<0) and risk elevation (SHAP>0) for COVID-19 outcomes. Mean SHAP values for high-contribution factors function as reference thresholds, facilitating the identification of factor-specific impact zones. This integrated analytical framework demonstrates both the direction and magnitude of factor effects while accounting for uncertainty in the estimated relationships.

### Ethical Considerations

This study was approved by the Institutional Review Board of Shantou University (approval number: STU202510002, approval date: October 28, 2025). The study exclusively uses publicly available, deidentified, aggregate-level epidemiological data from national and international health agencies, thereby not constituting human subjects research requiring individual informed consent. All data sources used provide data under open access licenses permitting academic research use. No individual-level patient data are collected, accessed, or analyzed; all metrics represent population-level aggregates, ensuring complete anonymization and elimination of privacy risks. The research involves no direct participant recruitment or clinical interventions, and no compensation was provided to participants. Data governance complies with institutional policies for secondary analysis of public health surveillance data, with no ethical concerns regarding confidentiality, consent, or participant identification.

## Results

### Global Contributions and Temporal Dynamics of COVID-19 Burden Drivers

**Figure 1:**
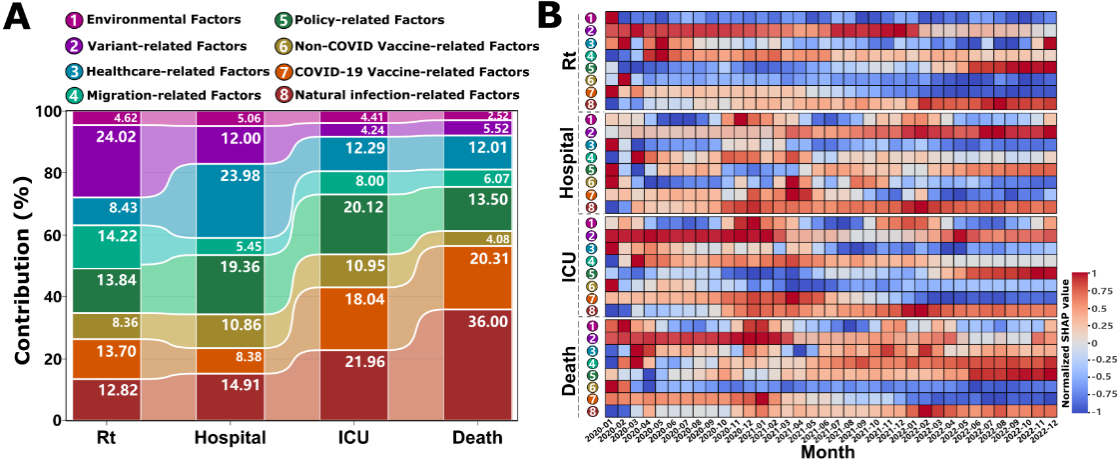
Contribution of factor groups to COVID-19 burden metrics during the pandemic. (A) Relative contributions of 8 factor groups to 4 COVID-19 burden metrics (Rt: effective reproduction number representing transmission potential; Hospital: daily new hospital admissions per million population; ICU: daily new intensive care unit admissions per million population; Death: daily COVID-19-attributed deaths per million population). Bars represent mean contribution percentages across all 38 countries and the entire study period (January 2020 to December 2022). (B) Temporal dynamics of normalized SHAP values for each factor group across the 4 outcomes. Each row corresponds to one of the 8 factor groups (identified by the same color and number as in Panel A). Heatmap cells represent monthly-aggregated normalized SHAP values (Z-score standardized within each factor group, range: –1 to +1), where red indicates positive contributions (promoting increased disease burden) and blue indicates negative contributions (suppressing disease burden).

Through comprehensive big data analytics and machine learning approaches, we delineate the differential contribution patterns among factor groups to various COVID-19 burden metrics (including transmission potential: Rt, hospitalizations, ICU admissions, and mortality rates) by implementing the XGBoost model in conjunction with SHAP values. The analytical framework encompasses SHAP importance analysis across the entire pandemic period (Figure 1A), while using the PCA algorithm to visualize the contributions and temporal evolution of each factor group (Figure 1B). The model’s performance metrics were rigorously validated through time-series cross-validation across all 38 countries (Tables S3 and S4 in Multimedia Appendix 2). Robustness analyses confirmed stable factor group contribution rankings across sensitivity scenarios (Figure S4 in Multimedia Appendix 2).

Our findings, illustrated in Figure 1A and Table S5 in Multimedia Appendix 2, show that the variant-related group has the highest contribution 24.02% (95% CI 10.10%-66.88%) to COVID-19 transmission (Rt), but its influence decreases dramatically with increasing disease severity (only 4.24% [95% CI 1.59%-10.89%] and 5.52% [95% CI 1.94%-15.39%] for ICU admissions and deaths, respectively). The immunity-related group, including natural infection-related, COVID-19 vaccine–related, and non-COVID vaccine-related factors, emerges as a consistently important contributor across all burden metrics. Specifically, the average contribution of natural infection-related factors showed an ascending pattern of contribution with increasing disease severity, progressively increasing from 12.82% for transmission and 14.91% for hospitalization to 21.96% and 36.00% of the variance in ICU admissions and deaths, respectively. Similarly, the COVID-19 vaccination–related factors maintain substantial contributions to severe outcomes (18.04%, 95% CI 6.39%-42.49% for ICU admissions and 20.31%, 95% CI 6.53%-58.31% for deaths), although its influence on transmission and hospitalization is relatively lower (13.70%, 95% CI 3.85%-35.51% and 8.38%, 95% CI 3.50%-22.29%, respectively). Notably, routine immunization (non–COVID-19 vaccines) demonstrated meaningful contributions (ranging from 4.08%, 95% CI 1.52%-11.48% to 10.95%, 95% CI 4.27%-53.05%) across all metrics, with particularly pronounced effects on hospitalization and ICU admissions. The health care–related group exerted the strongest influence on hospitalization outcomes (23.98%, 95% CI 7.03%-73.13%), where higher per capita GDP and nursing staff density bolstered resilience, albeit modulated by population size (Figure S5 in Multimedia Appendix 2). Policy interventions significantly shaped disease burden (Figure S6 in Multimedia Appendix 2), and migration factors substantially drove transmission dynamics (averaging 14.22% of Rt variation, Figure S7 in Multimedia Appendix 2), whereas environmental impacts were consistently modest (<5%, Figure 1A). Disaggregating these, age structure (proportion ≥65 years) showed minimal contributions to transmission (0.33%, 95% CI 0.01%-2.15%) and hospitalization (0.53%, 95% CI 0.02%-3.28%) but markedly higher for ICU admissions (1.55%, 95% CI 0.03%-9.96%) and mortality (1.72%, 95% CI 0.15%-6.00%), underscoring a distinct age-severity gradient. Conversely, health care infrastructure (including the number of doctors, nurses and pharmacists, medical input and beds) most affected hospitalization burden (12.54%, 95% CI 0.37%-48.88%), exceeding its influence on ICU (8.21%, 95% CI 0.28%-35.67%) and mortality (6.43%, 95% CI 0.19%-28.54%), indicating its primary role in modulating admission thresholds rather than altering ultimate severe outcomes (Table S5 in Multimedia Appendix 2).

Temporal epidemiological analysis reveals dynamic patterns in factor group influences throughout the pandemic (Figure 1B). For interpretative purposes, normalized SHAP values approaching 1 indicate strong positive contributions (promoting increased disease burden), while values nearing –1 suggest substantial negative contributions (suppressive effect on disease burden). The variant-related group generally exerts promoting effects on COVID-19 burden metrics, particularly during the initial outbreak phase (January 2020 to January 2021), as evidenced by increasing red intensity (Figure 1B). While the magnitude of these effects generally attenuates over time, Rt presents a notable exception, demonstrating persistently strong promoting effects from July 2021 to January 2022 (Figure 1B depicts the variant-related group for Rt). This period coincides with the global dominance of the highly transmissible Delta 21J and Omicron 21K variants. Subsequently, following the widespread implementation of mass vaccination programs globally (post-March 2021), COVID-19 vaccines demonstrate an immediate and pronounced suppressive effect on transmission. This protective immunity is sustained over time, reflected by increasingly negative SHAP values (blue intensity, Figure 1B, COVID-19 vaccine–related group for Rt). A one-to-three-month lag is observed before similar suppressive effects manifested across other burden metrics (Figure 1B, COVID-19 vaccine-related group for hospitalization, ICU admissions, and deaths, respectively). Non–COVID-19 vaccines consistently exhibit long-term protective effects (Figure 1B, non–COVID-19 vaccine–related group). Conversely, factors related to natural infection potentially contribute to an increased disease burden during the endemic phase of the pandemic (post-2022, Figure 1B depicts the natural infection-related group). Intriguingly, the environmental group demonstrates distinct seasonal patterns of influence on hospitalization, ICU admissions, and mortality rates (Figure 1B, environmental group for hospitalization, ICU admissions, and deaths, respectively). Specifically, these factors exhibit a propensity to promote increased disease burden during the winterspring seasons (November to March). Conversely, a protective influence is observed during the summer-autumn periods (April to September), suggesting a seasonal modulation of environmental risk factors.

### Impact of SARS-CoV-2 Variant Prevalence on Transmission Dynamics

Given that the variant-related group emerges as the predominant contributor to Rt (24.02%, 95% CI 10.10%-66.88%), shown in Figure 1A, we conduct a detailed analysis of how the prevalence of different variants influences COVID-19 transmission dynamics. We standardize the SHAP values of 13 major variants that have circulated during the pandemic to enable systematic comparison of their transmission potential.

**Figure 2:**
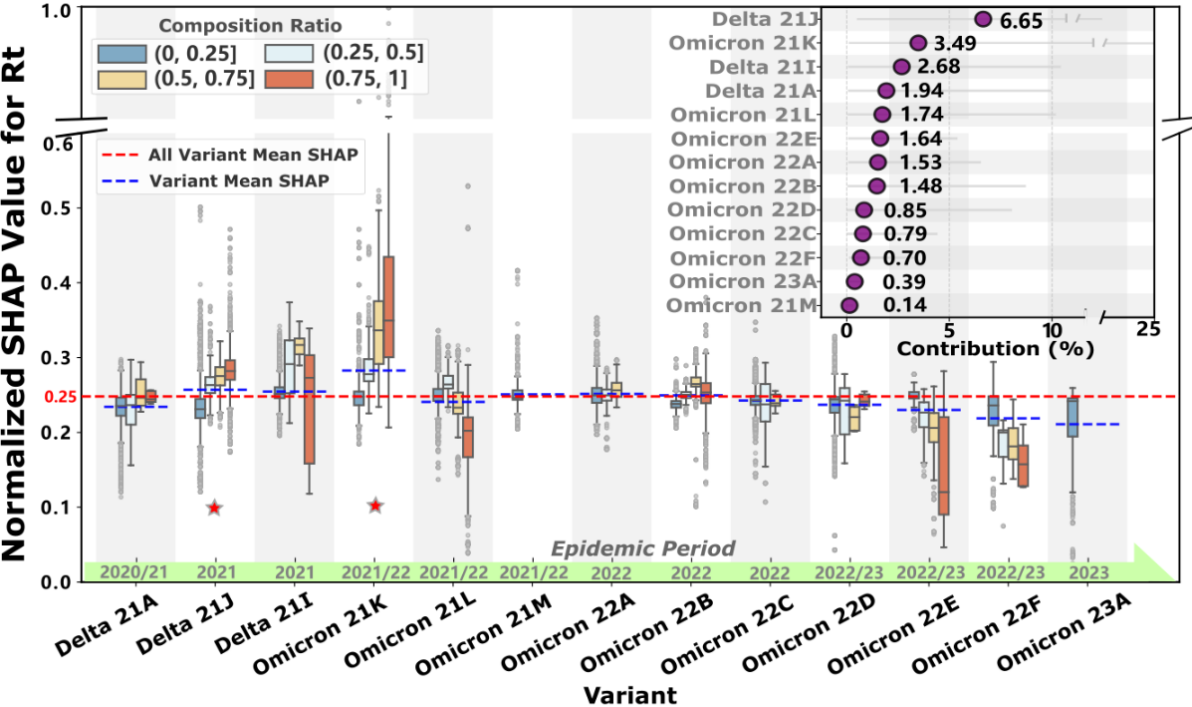
Variant composition ratio effects on COVID-19 transmission (Rt) across dominant variants during the pandemic. The main panel displays normalized SHAP values (y-axis, representing marginal contribution to Rt) for 13 major SARS-CoV-2 variants (x-axis, arrange from left to right in order of global popularity over time) across 4 composition ratio levels (color-coded box plots: dark blue = low ratio <25%, medium blue = medium-low ratio 25%-50%, light orange = medium-high ratio 50%-75%, dark orange = high ratio >75%). Each box plot summarizes the distribution of SHAP values across all country-day observations where the variant circulated at the specified composition ratio, with box boundaries representing 25th and 75th percentiles, central line indicating median, and whiskers extending to 1.5×IQR. The red dashed horizontal line indicates the overall mean SHAP value (0.25) across all variants and observations, serving as the baseline transmission intensity. The blue dashed horizontal lines represent variant-specific mean SHAP values. Variants marked with stars: Delta 21J and Omicron 21K demonstrate significant enhanced transmissibility at higher composition ratios, with mean SHAP values (0.26 and 0.28, respectively) exceeding the overall baseline. Inset panel (top right): Overall contribution of each variant to Rt variance across the entire study period, with error bars representing 95% CIs; variants are ordered identically to the main panel to facilitate comparison. Green timeline bar (bottom): Temporal circulation periods of major variants, indicating the years (2020-2022) when each variant achieved dominant prevalence (>50% composition ratio) in at least 10 countries, illustrating the sequential emergence and replacement dynamic. Rt: effective reproduction number; SHAP: Shapley Additive Explanation.

As shown in Figure 2, the baseline means transmission intensity (red line) across all variants is 0.25 (normalized SHAP value). Six lineages exceed this threshold: Delta (21J, 21I) and Omicron (21K, 21M, 22A, 22B). Notably, Delta 21J (contribution: 6.65%, 95% CI 0.49%-20.76%) and Omicron 21K (contribution: 3.49%, 95% CI 0.1%-25.22%) demonstrate stronger transmission potential with increasing prevalence (marked with asterisks in Figure 2). Their normalized SHAP values of 0.26 and 0.28, respectively, indicate transmission intensities 3.4% and 12.2% higher than the baseline. This finding aligns with the pronounced transmission peaks observed during their predominant circulation period (July 2021 to January 2022) as shown in Figure 1B. Interestingly, several variants were displaced by competing lineages before achieving widespread dominance (eg, Omicron 21M, 22A, and 23A). Meanwhile, some variants, such as Omicron 22E and 22F, exhibited weakening transmission potential with increasing prevalence during their dominance period (August to December 2022, Figure 1B), ultimately leading to their gradual replacement.

### Global Impact of Natural Infection and Immunization Factors on COVID-19 Burden Metrics

Building upon the overall factor importance analysis, we find that infection and immunization-related factors (including natural infection, COVID-19 vaccination, and non–COVID-19 immunization) are critical determinants of all COVID-19 burden metrics. Collectively, these factors average account for 34.88% to 60.39% of the total feature contribution, with a notably higher contribution for severe burden metrics such as ICU admissions and mortality (Figure 1A). To elucidate the specific effects of these key factors, we conduct SHAP dependence analyses to assess their nonlinear relationships with COVID-19 burden metrics.

**Figure 3:**
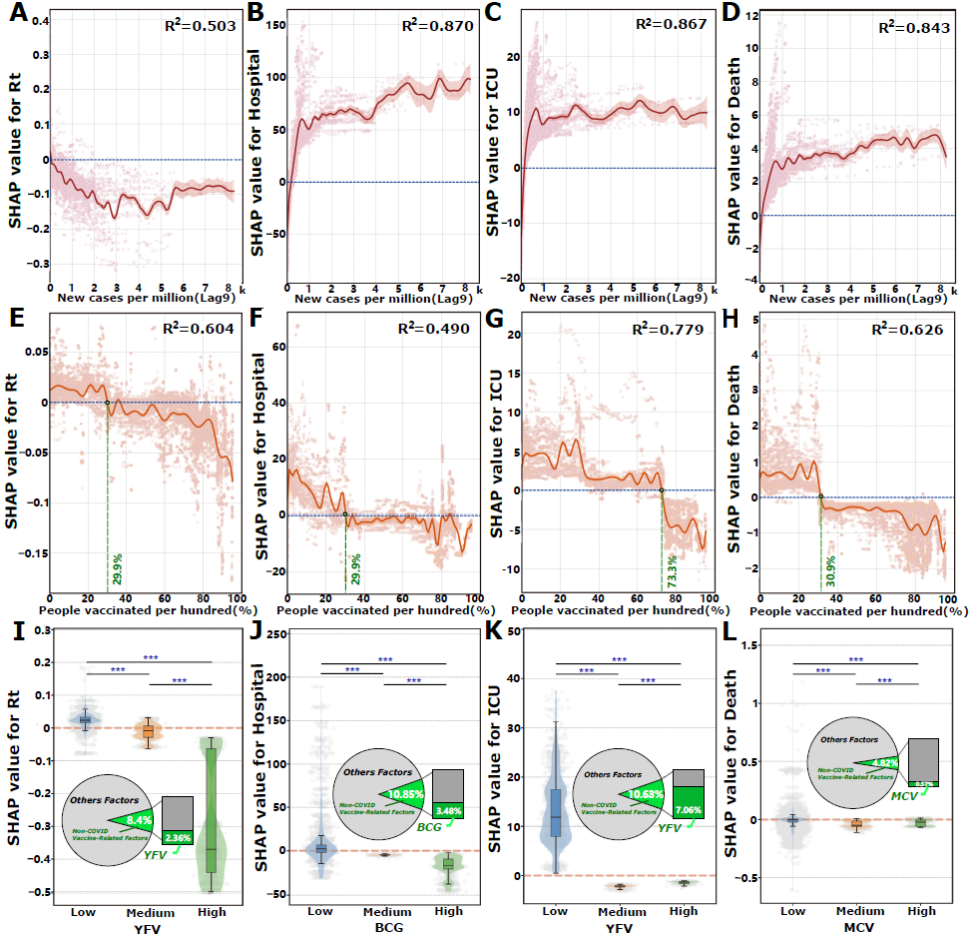
Effects of infection and immunization-related factors on COVID-19 burden. Panels A-D illustrate the impact of infection-related factors on various outcomes: the Rt in the population following new infections (A), hospital admissions (B), intensive care unit (ICU) admissions (C), and mortality (D). (E-H): The impact of vaccination coverage (%) of the population vaccinated on COVID-19 burden metrics. Vaccination exhibits a threshold effect (green dashed lines), beyond which it significantly reduces Rt (E), hospitalizations (F), ICU admissions (G), and mortality (H). The nonlinear relationships are fitted using a generalized additive model, with shaded regions representing 95% CIs, indicating the uncertainty of the fitted relationships. (I-L) The effects of non-COVID routine immunizations on COVID-19 burden metrics. The most impactful vaccines are presented: YFV vaccine for Rt (I) and ICU admissions (K), BCG vaccine for hospitalizations (J), and MCV vaccine for mortality (L). SHAP values < 0 (below the red dashed line) indicate suppressive effects on the respective metrics. The inset pie charts illustrate the relative contributions of these specific vaccines to the total effect of all non-COVID vaccines. BCG: Bacillus Calmette–Guérin; MCV: measles-containing vaccine; Rt: effective reproduction number; SHAP: Shapley Additive Explanation; YFV: yellow fever vaccine.

Following infection, the resultant immune protection demonstrates a lagged effect (Figure S1A and Figure S2A in Multimedia Appendix 2), effectively controlling the Rt growth (Figure 3A). This inhibitory effect intensifies with increasing numbers of new infections. Notably, this inhibitory effect becomes more pronounced with higher levels of both new and cumulative infections (Figure S8A in Multimedia Appendix 2), suggesting that both recent infection waves and accumulated population immunity can significantly disrupt subsequent transmission dynamics. In contrast, for more severe outcomes—hospitalization, ICU admissions (Figure 3B/C), and mortality (Figure 3D)—new infections amplify the burden. For mortality, the relationship is particularly striking: higher levels of new infections consistently exacerbate fatal outcomes (Figure 3D). Regarding cumulative infection rates, ICU admissions exhibit a strong positive association when the cumulative infection rate is below 10% (ie, fewer than 100,000 cases per million people), indicating a rapid escalation in ICU admissions during early outbreak phases (Figure S8C in Multimedia Appendix 2). Beyond this threshold, the effect on ICU burden is more moderate, albeit still positive. For mortality (Figure S8D in Multimedia Appendix 2), while a similar pattern is observed, cumulative infection rates exceeding 50% appear to reduce mortality burdens, as indicated by SHAP values below the reference line. This finding implies potential population-level protective effects, possibly attributed to acquired immunity in highly infected populations.

From an immunization perspective, increasing the proportion of the population vaccinated consistently exerts a suppressive effect on all burden metrics (Figures 3E-H). This relationship is nonlinear and exhibits threshold effects, wherein surpassing specific vaccination rates enhances the efficacy of burden reduction. The identification of these critical vaccination thresholds is grounded in SHAP-based risk decision boundary analysis: threshold values are defined as the vaccination coverage levels where the GAM-fitted SHAP curve crosses zero (transition from positive to negative marginal effects on disease burden), with statistical significance confirmed by nonoverlapping 95% CIs before and after the threshold point. Specifically, vaccination rates of 29.9% (95% CI 29.8%-29.9%) suppress Rt, 29.9% (95% CI 29.4%-31.4%) reduce hospitalization rates, 72.3% (95% CI 72.2%-72.8%) diminish ICU admissions, and 30.9% (95% CI 30.7%-31.1%) decrease mortality. Additionally, we assess the impacts of complete vaccination and booster doses on COVID-19 burden metrics (Figure S9 in Multimedia Appendix 2).

Our analysis reveals that certain non–COVID-19 routine immunizations, particularly those with high contribution scores, are found to suppress COVID-19 burdens, especially when administered at medium to high doses. For instance, high-dose administration of the YFV (>600,000 doses) significantly reduces Rt (Figure 3I). A similar dose-response relationship is observed for ICU admissions, where YFV doses exceeding 100,000 (medium or high dose levels) exhibit a protective effect (Figure 3K). For other burden metrics, BCG vaccination exceeding 450,000 doses leads to a modest reduction in hospitalizations (Figure 3J), while the measles-containing vaccine administration exceeding 10 million doses reduces mortality burdens (Figure 3L). The statistical significance of these dose-based thresholds is confirmed using Mann-Whitney *U* tests (*P*<.01).

### Seasonal Modulation of Environmental Factors on COVID-19 Burden Metrics

Our previous analysis reveals that the composite effect of environmental factors, derived via PCA on multivariate environmental time-series data, exhibits heterogeneous patterns of promotion and inhibition across the majority of COVID-19 burden metrics (Figure 1B). Notably, despite these observable periodic influences, the majority contribution of the environmental group remains below 5%. To elucidate the determinants underlying the heterogeneous risk modulation induced by environmental factors, we used SHAP local dependency analysis. Our findings highlight that temperature emerges as a regulatory factor, with its overall contribution accounting for 1.37% (95% CI 0.06%-5.73%) and 1.59% (95% CI 0.14%-4.42%) of the variance in hospital and ICU admissions, respectively. In contrast, humidity contributed 1.46% (95% CI 0.07%-5.51%) and 0.29% (95% CI 0.008%-1.18%) to hospital and ICU admissions, respectively (Figure S3 and Table S5 in Multimedia Appendix 2).

**Figure 4:**
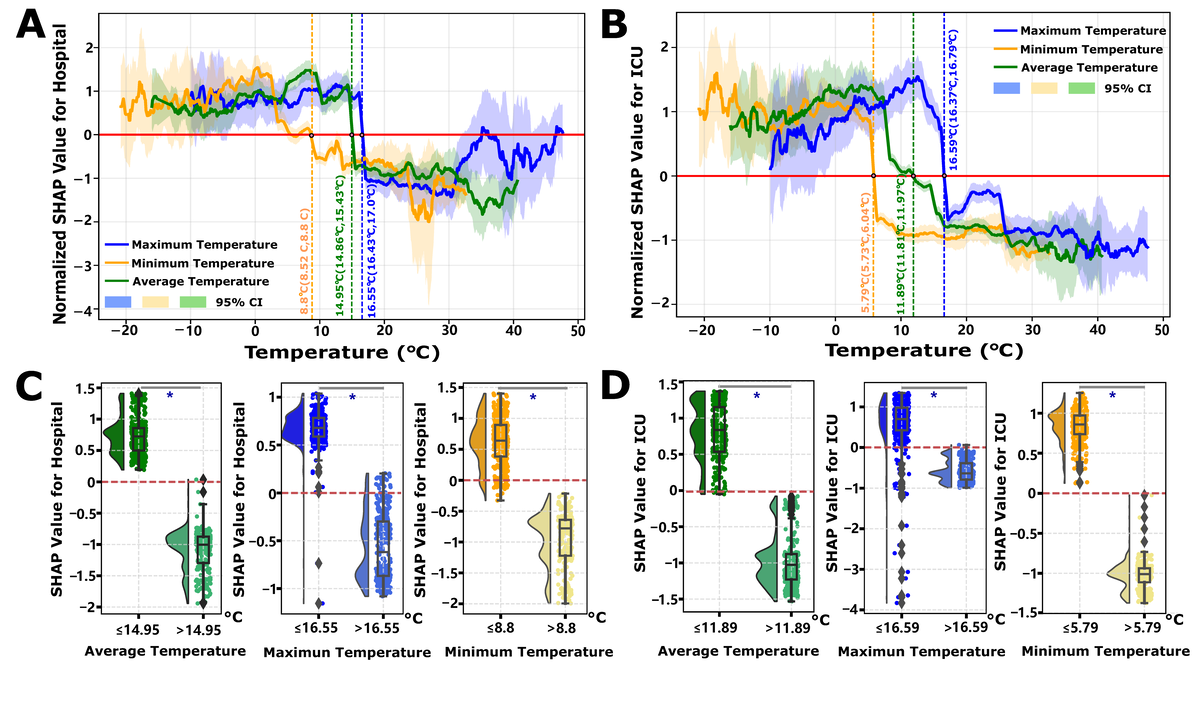
Impact of temperature on COVID-19 hospitalization and ICU admissions. (A) Effects of temperature variables (mean, minimum, and maximum temperatures) on hospitalization burden. (B) Effects of temperature variables on severe case burden. SHAP values are standardized within groups using Z-scores, with SHAP = 0 serving as the control line indicating the point of risk decision shift. (C) Temperature threshold analysis showing risk decision surface differences for hospitalization burden. (D) Temperature threshold analysis showing risk decision surface differences for severe case burden. *P<.05 indicates statistically significant threshold differences.

Leveraging extensive temporal environmental data, a more granular examination of temperature reveals the existence of distinct risk decision boundaries. The determination of temperature thresholds follows a SHAP-based risk transition framework: for each temperature variable (mean, minimum, and maximum), we fit GAM curves to SHAP values across the observed temperature range and identify the threshold as the temperature value where SHAP=0, representing the decision boundary between risk elevation (SHAP > 0, promoting disease burden) and risk reduction (SHAP<0, suppressing burden). Statistical significance is confirmed by nonoverlapping 95% CIs of SHAP values in the temperature ranges immediately below versus above the threshold (*P*<.05 via bootstrap resampling). Specifically, lower temperatures are associated with an increased burden of both hospital and ICU admissions, whereas higher temperatures appear to confer a protective effect, mitigating these burdens. The analysis identifies precise temperature thresholds acting as risk transition points: for hospital admissions, the temperature threshold is determined to be 14.95°C (with a minimum and maximum temperature ranging from 8.8°C to 16.55°C, Figure 4A), and for ICU admissions, the threshold is identified at 11.89°C (ranging from 5.79°C to 16.59°C, Figure 4B). Importantly, the determination of these temperature thresholds—encompassing average, maximum, and minimum temperatures—demonstrates statistically significant differences before and after the threshold points (*P*<.05, Figure 4C/4D). Continental stratification analyses confirmed similar temperature-dependent risk transition patterns across 5 continents, with region-specific decision boundaries for both hospitalization and ICU admissions (Figures S10 and S11 in Multimedia Appendix 2), validating the global threshold findings. Humidity exhibits a more complex influence on COVID-19 burden metrics. For hospital admissions, absolute humidity displays a U-shaped relationship, indicating that values outside the range of 8-16 g m^–3^ are associated with an increased risk (Figure S12A in Multimedia Appendix 2). Conversely, for ICU admissions, absolute humidity exceeding 18 g m^–3^ is linked to a heightened risk (Figure S12B in Multimedia Appendix 2). Contrary to temperature, humidity does not exhibit analogous risk threshold decision points concerning the Rt and mortality burdens (Figure S13A,B in Multimedia Appendix 2). Nonetheless, moderate temperature conditions (average temperatures above 15°C for Rt (Figure S13C in Multimedia Appendix 2) and above 5°C for mortality (Figure S13D in Multimedia Appendix 2) are associated with reduced risks. In these scenarios, the impact of humidity is negligible, particularly concerning mortality, where SHAP values for humidity remain close to zero, manifesting as a near-constant line (Figure S14 in Multimedia Appendix 2).

## Discussion

### Principal Findings

This study presents a multicountry, time-resolved analysis of multifactorial COVID-19 burden determinants across 38 nations, addressing the research questions and hypotheses posed in the Introduction through big data analytics integrated with interpretable machine learning. Findings are consistent with all four a priori hypotheses: (1) variants most strongly shape transmission (Rt), with attenuated influence on severe outcomes; (2) population immunity displays threshold-dependent effects that differ for transmission versus severity; (3) selected routine immunizations show dose-response associations consistent with cross-protective “trained immunity”; and (4) environmental factors modulate burden through nonlinear thresholds. Beyond hypothesis testing, 3 underrecognized patterns emerge: variant “fitness” appears shaped by contemporaneous immunity landscapes rather than intrinsic transmissibility alone; a hierarchical immunity architecture requires higher coverage to prevent severe outcomes than to reduce transmission; and population-level trained immunity from routine vaccines may contribute to resilience. Together, large-scale, multidimensional time-series data clarify how viral evolution, immunity dynamics, and environment jointly structure COVID-19 burden, yielding interpretable, hypothesis-generating metrics for preparedness [54].

Variants dominate transmission (24.02% contribution to Rt, 95% CI 10.10%-66.88%) but attenuate toward severe outcomes (4.24% for ICU, 5.52% for deaths; Figure 1A), with Delta 21J and Omicron 21K exhibiting 3.4% and 12.2% higher transmissibility than baseline (Figure 2). This differential contribution pattern reveals a previously overlooked mechanism: variant evolutionary success is shaped not solely by intrinsic biological properties but by dynamic interactions with population immunity landscapes [55]. The displacement of Omicron sublineages (21M, 22A, 23A) before achieving dominance, and the declining transmission potential of 22E/22F despite high initial prevalence (Figure 1B, August-December 2022), suggests that viral evolution follows a “path of least immunological resistance”—variants must balance immune escape capacity with transmission efficiency in heterogeneous immunity contexts [56,57]. This pattern aligns with recent AI-driven variant prediction models using ensemble methods and deep learning fusion approaches for COVID-19 detection [58,59], yet our SHAP-interpreted framework advances beyond black-box predictions by quantifying temporal threshold dynamics and revealing variant-immunity co-evolution [60]. Mechanistically, the temporal lag in variant effects (strongest during January 2020-January 2021, Figure 1B) likely reflects initial population immunological naivety, whereas subsequent attenuation corresponds to accumulating hybrid immunity from infection and vaccination.

Conversely, natural infection contributions escalate with severity (12.82% for Rt to 36.00% for deaths, 95% CI 10.25%-78.56%; Figure 1A), exhibiting dual roles: protective against transmission via lagged immunity (Figure 3A) but amplifying severe burdens during early outbreaks (Figures 3B-D). This dichotomy arises from heterogeneous immune waning and reinfection vulnerabilities in populations with incomplete immunity coverage [61]. The ICU threshold at 10% cumulative infection rate (Figure S8C in Multimedia Appendix 2) suggests a critical tipping point where health care systems transition from manageable to crisis states, while mortality reduction beyond 50% infection rate (Figure S8D in Multimedia Appendix 2) implies population-level protective effects emerging only after widespread exposure—a pattern potentially reflecting trained innate immunity or T-cell cross-reactivity from endemic coronavirus exposures [62]. Health care infrastructure’s predominant role in hospitalization (23.98%, 95% CI 7.03%-73.13%; Figure 1A and Figure S4 in Multimedia Appendix 2) underscores resource-dependent outcome modulation, where capacity acts as a buffer against admission surges rather than altering ultimate severity—a critical distinction for health care planning.

Vaccination demonstrates hierarchical protection with strikingly different thresholds: 29.9% coverage (95% CI 29.8%-29.9%) suppresses Rt/hospitalization/mortality, while 72.3% (95% CI 72.2%-72.8%) is required for ICU prevention (Figures 3E-H). This tiered architecture reveals a previously unrecognized immunological insight: vaccine-induced immunity operates through distinct mechanistic pathways across disease severity gradients. Lower coverage likely achieves transmission reduction via humoral antibody responses providing transient infection blockade, whereas higher coverage is necessary for severe disease prevention through durable cellular immunity (T-cell mediated pathology mitigation) [57,61,62]. This hypothesis is supported by the 1-3 month temporal lag before protective effects emerge across severity metrics (Figure 1B), corresponding to T-cell response maturation timescales [62]. Compared with prior AI-driven vaccination modeling using SHAP-interpreted ensemble methods, our framework uniquely identifies dual coverage thresholds with temporal resolution, advancing beyond aggregate vaccination effects to reveal severity-specific immunity requirements [63]. Most intriguingly, routine immunizations exhibit dose-specific cross-protection: YFV >600,000 doses reduces Rt (Figure 3I) and >100,000 doses protect against ICU (Figure 3K), while BCG >450,000 doses reduces hospitalizations (Figure 3J). These dose-response patterns suggest population-level trained immunity effects—where diverse pathogen exposures prime innate immune memory (eg, epigenetic reprogramming of monocytes/macrophages), conferring nonspecific protection against SARS-CoV-2 [64,65]. However, this cross-protection hypothesis requires mechanistic validation through immunological assays (eg, cytokine profiling and immune cell phenotyping), as our observational design cannot exclude confounding by health care access or socioeconomic factors correlated with routine immunization coverage [66,67].

Environmental factors demonstrate nonlinear threshold effects: temperatures below 14.95°C (95% CI 14.86°C-15.43°C) increase hospitalizations, and below 11.89°C (95% CI 11.81°C-11.97°C) increase ICU admissions (*P*<.05; Figure 4), while humidity shows U-shaped relationships (optimal 8-16 g m^–3^; Figure S12A in Multimedia Appendix 2). These thresholds likely define “vulnerability windows” where low temperatures enhance viral aerosol stability and suppress mucosal immunity (reduced interferon responses), compounded by indoor crowding behaviors [68,69]. Yet environmental contributions remain modest (<5% overall, Figure 1A), suggesting they modulate disease timing rather than ultimate severity [70,71].

Several important limitations temper the interpretation of these findings. First, the ecological observational design identifies associations rather than causal relationships. Vaccination thresholds, environmental effects, and cross-protection from routine immunizations represent correlative patterns that may be confounded by unmeasured factors and require experimental validation through mechanistic immunological studies (eg, controlled vaccine trials, in vitro immune profiling) before inferring causation. Second, data biases compromise generalizability: sample selection of 38 high-burden countries, while representing >60% both of the global population and COVID-19 burden, systematically favors nations with robust surveillance infrastructure, potentially limiting applicability to low-resource settings with different reporting capacities; post-2022 reporting heterogeneity (discontinuation of systematic surveillance in many nations) creates missing data bias; and variant sequencing coverage variations across countries mean proportions may not fully represent circulating strains in undersequenced regions. Third, time-dependent confounding and nonstationarity in pandemic data pose threats to validity—reporting practices, testing intensity, and clinical definitions evolved over the study period, potentially introducing spurious temporal trends. While our partial autocorrelation function–based lag optimization mitigates some temporal biases, residual immortal time bias may persist in time-series analyses tracking outcomes over extended periods [72], and we cannot fully exclude confounding from unconsidered time-varying factors (eg, behavioral changes and antiviral treatment availability). Fourth, factor interactions were not explicitly modeled; the XGBoost framework captures some nonlinear interactions, but synergistic or antagonistic effects between variants, immunity, and environmental factors remain incompletely characterized. Fifth, identified thresholds (vaccination coverage and temperature) likely vary across geographic/demographic contexts due to differences in population behavior, health care infrastructure, and climatic conditions, limiting global applicability without region-specific validation. Finally, the study lacks external replication with independent datasets from different countries or time periods post 2022, essential for confirming reproducibility and establishing reliability before the framework can be considered a generalizable template for pandemic response.

Priority next steps include (1) external validation using post 2022 independent datasets to test threshold stability and replication in low-resource settings, (2) mechanistic validation of trained immunity through randomized trials and immunological assays measuring innate immune markers, (3) integration of additional predictors (host genetics, viral genomic features, and social determinants), (4) methodological refinements addressing temporal biases (time-varying models and landmark analysis), and (5) operationalization into real-time decision support tools translating thresholds into automated public health alerts.

### Conclusions

This study demonstrates how interpretable machine learning applied to large-scale, multidimensional pandemic surveillance data can systematically decompose complex burden determinants into quantifiable contributions and actionable thresholds. Three underrecognized population-level patterns emerge: variant evolutionary trajectories constrained by shifting immunity landscapes rather than intrinsic fitness accumulation, hierarchical immunity architecture requiring differential coverage targets across the severity spectrum, and potential trained immunity from routine immunization programs contributing to pandemic resilience. At the same time, the observational design limits causal inference; the integration of viral genomic surveillance, immunization records, environmental monitoring, and clinical outcomes within a unified analytical framework generates mechanistic hypotheses prioritizing experimental validation. This approach offers a methodological template for rapid multifactorial analysis in future epidemics, provided findings undergo pathogen-specific adaptation and independent replication to establish reproducibility and guide evidence-based preparedness strategies.

## Appendix

Multimedia Appendix 1 Critical Appraisal Skills Programme (CASP) checklist.

Multimedia Appendix 2 Supplementary materials.

Multimedia Appendix 3 Contribution of factors and factor groups to COVID-19 burden metrics.

## Abbreviations

AI: artificial intelligence
BCG: Bacillus Calmette–Guérin
GAM: generalized additive model
ICU: intensive care unit
NPI: nonpharmaceutical intervention
PCA: principal component analysis
Rt: effective reproduction number
SHAP: Shapley Additive Explanation
UNICEF: United Nations Children's Fund
WHO: World Health Organization
XGBoost: extreme gradient boosting
YFV: yellow fever vaccine

## Data Availability

The data that support the findings of this study are available from the corresponding author upon reasonable request.
