# Supplementary material for: Systematic Determinants of Global COVID-19 Burden: Longitudinal Time-Series Analysis Using Big Data-Driven Artificial Intelligence
Source: J Med Internet Res. 2025 Dec 29;27:e79745. doi: 10.2196/79745 (PMC12796881; doi:10.2196/79745)
Supplement: Multimedia Appendix 2 [file jmir_v27i1e79745_app2.docx]

**Supplementary Materials for**

**Systematic Determinants of Global COVID-19 Burden: Longitudinal Time-Series Analysis Using Big Data-Driven Artificial Intelligence**

Zicheng Cao^1,2,3^, Wenjie Han^2,3^, Xue Zhang^2,3^, Chi Zhang^2,3^, Jinfeng Zeng^2,3,4^, Yilin Chen^2,3^, Haoyu Long^2,3,5^, Jian Chen^2,3^, Xiangjun Du^2,3,6,7^*

^1^School of Public Health, Shantou University, Shantou 515041, P.R. China

^2^School of Public Health (Shenzhen), Shenzhen Campus of Sun Yat-sen University, Shenzhen 518107, P.R. China

^3^School of Public Health (Shenzhen), Sun Yat-sen University, Guangzhou 510275, P.R. China

^4^School of Public Health, Fudan University, Key Laboratory of Public Health Safety, Ministry of Education, Shanghai, 200032, China

^5^Guangdong Provincial Center for Disease Control and Prevention, Guangzhou, 511430, China

^6^Key Laboratory of Tropical Disease Control, Ministry of Education, Sun Yat-sen University, Guangzhou 510030, P.R. China

^7^Shenzhen Key Laboratory of Pathogenic Microbes & Biosafety, Shenzhen Campus of Sun Yat-sen University, Shenzhen 518107, P.R. China

***Corresponding to:**

Dr. Xiangjun Du

School of Public Health (Shenzhen), Sun Yat-sen University

No.66, Gongchang Road, Guangming District Shenzhen, Guangdong 518107, P. R. China

Email: duxj9@mail.sysu.edu.cn

**Appendices**

**Table S1:** The 38 Countries incorporated in the study

**Table S2:** Description of policy-related factors

**Table S3:** Performance evaluation of XGBoost model on time-series datasets of COVID-19 burden metrics

**Figure S1:** Determination of lag periods for time-dependent factors influencing COVID-19 metrics

**Figure S2:** Identification of optimal lag periods for cumulative infections in influencing COVID-19 burden metrics

**Figure S3:** Contribution of factors to COVID-19 burden metrics

**Figure S4:** Impact of Healthcare-Related Group on Hospitalization Burden

**Figure S5:** Impact of the Top Three Influential NPIs on COVID-19 Disease Burden, as measured by their implementation stringency

**Figure S6:** Impact of International, Domestic Air Travel (A, C) and Their Lag Periods (B, D) on the Rt of COVID-19

**Figure S7:** Nonlinear Effects of Cumulative Natural Infections on COVID-19 Burden Metrics

**Figure S8:** Nonlinear effects of full vaccination and booster vaccination rates (per hundred, %) on severe COVID-19 burden metrics

**Figure S9:** Effects of Absolute Humidity Range on (A) Hospitalizations and (B) ICU Admissions

**Figure S10:** Dynamic Effects of Temperature Factors on COVID-19

**Figure S11:** Dynamic Effects of Humidity Factors on COVID-19

**Materials & Methods**

**Policy index.** The classification variables originally used to highlight the intensities of policy implementation try now to be more illustratively expressed by transmuting it into a policy score. This necessitates the application of a specific mathematical formula intended to accomplish this transformation as into policy scores. The formula,

$$I_{j, t}=100\times\frac{v_{j,t}-0.5\times(F_{j}-f_{j,t})}{N_{j}}$$

applies to each sub-policy score ($I$) associated with a particular indicator ($j$) on a specific day (*t*). In this formulation,$N_{j}$ denotes the maximum possible value of the indicator ($j$). The indicator $j$ accommodates a flag variable ($F_{j}$) that can take values of 1 or 0, indicating the presence or absence of the said flag variable, respectively. The elements $v_{j,t}$ and$f_{j,t}$ correspond to the documented policy value on the ordinal scale and the documented binary flag for the indicator $j$, respectively. The conversion of distinct classification variables yields a sub-policy score that ranges between 0 to 100, where each full point on the ordinal scale is equally spaced.

**Hyperparameter Optimization.** The model optimization process incorporates Bayesian hyperparameter techniques combined with time-series cross-validation, enabling the identification of optimal hyperparameters and enhanced model performance. Specifically, we employ the Optuna framework (version 3.1.0) to conduct Bayesian optimization via Tree-structured Parzen Estimator (TPE) sampling across the following hyperparameter space:

- Learning rate (eta): [0.01, 0.3], log-uniform sampling

- Maximum tree depth: [3, 10], integer uniform

- Minimum child weight: [1, 10], integer uniform

- Subsample ratio: [0.6, 1.0], uniform

- Column sampling by tree: [0.6, 1.0], uniform

- L1 regularization (alpha): [0, 1.0], uniform

- L2 regularization (lambda): [0, 1.0], uniform

The optimization objective is to maximize *R²* on the validation set, with 100 trials conducted per outcome variable. All hyperparameter searches employ a fixed random seed (42) to ensure reproducibility.

**Bootstrap Validation of Factor Group Importance Rankings.** To assess the robustness of SHAP-based factor group importance estimates to sampling variability, we performed bootstrap resampling analysis for each COVID-19 burden outcome. In each of 1000 bootstrap iterations, we randomly resampled 80% of country-day observations with replacement, recalculated SHAP values using the trained XGBoost model with TreeExplainer (interventional perturbation), and aggregated absolute SHAP values within eight predefined factor groups (Natural Infection, Variant, Non-COVID Vaccine, COVID-19 Vaccine, Policy, Healthcare, Environmental, Migration). Group-level importance values were normalized to sum to 1.0 within each iteration. From the resulting bootstrap distributions, we computed mean importance, 95% confidence intervals (2.5th-97.5th percentiles), standard deviation, and coefficient of variation (CV = SD/mean) to quantify ranking stability. CV < 0.15 indicates high stability, 0.15-0.30 moderate stability, and >0.30 unstable estimates. Narrow confidence intervals and low CV values confirm that SHAP importance rankings are stable across temporal and geographic resampling, validating the robustness of factor contributions reported in main analyses (Supplementary Figure S4).

**SHAP Aggregation Methodology.** To derive interpretable summary metrics from the high-dimensional SHAP output (65 features × 38908 country-day observations × 4 outcomes), we employ a hierarchical aggregation strategy: (1) Temporal aggregation: For each country and feature, SHAP values are averaged across all time points to obtain a country-specific feature importance score. These temporal averaging smooths day-to-day fluctuations while preserving cross-national heterogeneity. (2) Absolute importance: Global feature importance is quantified as the mean of absolute SHAP values across all countries and time points, providing a measure of average magnitude of effect regardless of direction. (3) Directional effects: To assess whether features predominantly promote or suppress burden, we calculate the mean signed SHAP value, with positive values indicating net burden-promoting effects and negative values indicating net burden-suppressive effects. (4) Uncertainty quantification: 95% confidence intervals for importance and effect metrics are derived via bootstrapping (1,000 resamples at the country level), accounting for cross-national heterogeneity.

**Results**

**Table S1. The 38 Countries incorporated in the study**

| Area | Number | Country |
| --- | --- | --- |
| Asia | 8 | Bangladesh, China, Japan, Pakistan, South Korea, Philippines, Indonesia, India |
| Europe | 15 | Austria, Bulgaria, Denmark, Finland, France, Greece, Sweden, Italy, Iceland, Ireland, Spain, Germany, Norway, Portugal, Romania |
| Africa | 4 | Algeria, Cameroon, South Africa, Kenya, |
| North America | 3 | USA, Canada, Mexico |
| South America | 6 | Bolivia, Chile, Colombia, Argentina, Ecuador, Panama |
| Oceania | 2 | Australia, New Zealand |
| Total | 38 |  |

**Table S2. Description of policy-related factors**

| [Non-pharmaceutical intervention](http://www.baidu.com/link?url=-lmNTNX3hdd3UUDJ18Wiut7q14JzsBztkEaNj7ulbyJVWZqK-wOYqB9KzaiMkUKD6Uu84kefgeVxOlbL_hwMbzAwifT9tg2our67fUydtFjh9byiRiy4l0DzABSzamTf7wyeZt4sIYv7W2EtvBjSna) | Scale | Score | Description |
| --- | --- | --- | --- |
| *School closing* (Record closings of schools and universities.) | 0 | 0.00 | No measures |
|  | 1 | 16.67, 33.33 | Recommend closing |
|  | 2 | 50.00, 66.67 | Required closing (only some levels or categories, e.g. just high school, or just public schools) |
|  | 3 | 83.33, 100.00 | Required closing all levels |
| *Workplace closing* (Record closings of workplaces.) | 0 | 0.00 | No measures |
|  | 1 | 16.67, 33.33 | Recommend closing (or recommend work from home) |
|  | 2 | 50.00, 66.67 | Required closing (or work from home) for some sectors or categories of workers |
|  | 3 | 83.33, 100.00 | Required closing (or work from home) for all but essential workplaces (e.g. grocery stores, doctors) |
| *Cancel public events* (Record cancelling public events.) | 0 | 0.00 | No measures |
|  | 1 | 25.00, 50.00 | Recommend cancelling |
|  | 2 | 75.00, 100.00 | Required cancelling |
| *Restrictions on gatherings* (Record limits on private gatherings.) | 0 | 0.00 | No measures |
|  | 1 | 25.00 | Restrictions on very large gatherings (the limit is above 1000 people) |
|  | 2 | 37.50, 50.00 | Restrictions on gatherings between 101-1000 people |
|  | 3 | 62.50, 75.00 | Restrictions on gatherings between 11-100 people |
|  | 4 | 87.50, 100.00 | Restrictions on gatherings of 10 people or less |
| *Close public transport* (Record closing of public transport.) | 0 | 0.00 | No measures |
|  | 1 | 25.00, 50.00 | Recommend closing or significantly reduce volume, route and means of transport available. |
|  | 2 | 75.00, 100.00 | Required closing or prohibit most citizens from using it. |
| *Stay at home requirements* (Record orders to "shelter-in-place" and otherwise confine to the home.) | 0 | 0.00 | No measures |
|  | 1 | 16.67, 33.33 | Recommend not leaving house |
|  | 2 | 50.00, 66.67 | Required not leaving house with exceptions for daily exercise, grocery shopping, and 'essential' trips. |
|  | 3 | 83.33 | Required not leaving house with minimal exceptions (e.g. allowed to leave once a week, or only one person can leave at a time, etc.) |
| *Restrictions on internal movement* (Record restrictions on internal movement between cities or regions.) | 0 | 0.00 | No measures |
|  | 1 | 25.00, 50.00 | Recommend not to travel between regions or cities |
|  | 2 | 75.00, 100.00 | Internal movement restrictions in place |
| *International travel controls* (Record restrictions on international travel.) | 0 | 0.00 | No measures |
|  | 1 | 25.00 | Screening arrivals |
|  | 2 | 50.00 | Quarantine arrivals from some or all regions |
|  | 3 | 75.00 | Ban arrivals from some regions |
|  | 4 | 100.00 | Ban on all regions or total border closure |
| *Income support* (Record if the government is providing direct cash payments to people who lose their jobs or cannot work. This policy only includes payments to firms if explicitly linked to payroll and salaries.) | 0 | 0.00 | No income support |
|  | 1 | 25.00, 50.00 | Government is replacing less than 50% of lost salary or if a flat sum, it is less than 50% median salary. |
|  | 2 | 75.00, 100.00 | Government is replacing 50% or more of lost salary or if a flat sum, it is greater than 50% median salary. |
| *Debt or contract relief* (Record if the government is freezing financial obligations for households.) | 0 | 0.00 | No debt or contract relief |
|  | 1 | 50.00 | Narrow relief, specific to one kind of contract |
|  | 2 | 100.00 | Broad debt or contract relief |
| *Public information campaigns* (Record presence of public info campaigns.) | 0 | 0.00 | No Covid-19 public information campaign |
|  | 1 | 50.00 | Public officials urging caution about COVID-19 |
|  | 2 | 75.00, 100.00 | Coordinated public information campaign (e.g. across traditional and social media) |
| *Testing policy* (Record government policy on who has access to testing. This records policies about testing for current infection (PCR tests) not testing for immunity, such as antibody test.) | 0 | 0.00 | No testing policy |
|  | 1 | 33.33 | Only those who both (a) have symptoms and (b) meet Specific criteria (e.g. key workers, admitted to hospital, encountered a known case, returned from overseas) |
|  | 2 | 66.67 | Testing of anyone showing COVID-19 symptoms |
|  | 3 | 100.00 | Open public testing (e.g. "drive through" testing available to asymptomatic people) |
| *Contact tracing* (Record government policy on contact tracing after a positive diagnosis.) | 0 | 0.00 | No contact tracing |
|  | 1 | 50.00 | Limited contact tracing; not done for all cases |
|  | 2 | 100.00 | Comprehensive contact tracing; done for all identified cases |
| *Facial coverings* (Record policies on the use of facial coverings outside the home) | 0 | 0.00 | No policy |
|  | 1 | 12.50, 25.00 | Recommended |
|  | 2 | 37.50, 50.00 | Required in some specified shared/public spaces outside the home with other people present, or some situations when social distancing not possible |
|  | 3 | 62.50, 75.00 | Required in all shared/public spaces outside the home with other people present or all situations when social distancing not possible |
|  | 4 | 87.50, 10.00 | Required outside the home at all times regardless of location or presence of other people |
| *Vaccination policy* (Record policies for vaccine delivery for different groups) | 0 | 0.00 | No availability |
|  | 1 | 20.00 | Availability for ONE of following: key workers/ clinically vulnerable groups (non elderly) / elderly groups |
|  | 2 | 40.00 | Availability for TWO of following: key workers/ clinically vulnerable groups (non elderly) / elderly groups |
|  | 3 | 60.00 | Availability for ALL of following: key workers/ clinically vulnerable groups (non elderly) / elderly groups |
|  | 4 | 80.00 | Availability for all three plus partial additional availability (select broad groups/ages) |
|  | 5 | 100.00 | Universal availability |
| *Protection of elderly people* (Record policies for protecting elderly people) | 0 | 0.00 | no measures |
|  | 1 | 16.67, 33.33, 50.00 | Recommended isolation, hygiene, and visitor restriction measures in LTCFs and/or elderly people to stay at home |
|  | 2 | 66.67, 75.27 | Narrow restrictions for isolation, hygiene in LTCFs, some limitations on external visitors and/or restrictions protecting elderly people at home |
|  | 3 | 83.33, 100 | Extensive restrictions for isolation and hygiene in LTCFs, all non-essential external visitors prohibited, and/or all elderly people required to stay at home and not leave the home with minimal exceptions |

**Table S3. Performance evaluation of XGBoost model on time-series datasets of COVID-19 burden metrics**

| COVID-19 burden metrics | *MAE* | *RMSE* | *R^2^* |
| --- | --- | --- | --- |
| *Rt* | 0.063 | 0.087 | 0.939 |
| *Hospital* | 7.120 | 10.787 | 0.995 |
| *ICU* | 0.853 | 1.313 | 0.997 |
| *Death* | 0.180 | 0.322 | 0.985 |

**Table S4. Time series cross-validation performance of XGBoost models for COVID-19 burden metrics**

| COVID-19 burden metrics | *MAE* | *RMSE* | *R^2^* |
| --- | --- | --- | --- |
| *Rt* | 0.417 | 0.508 | 7.990 |
| *Hospital* | 70.271 | 84.088 | 21.238 |
| *ICU* | 10.881 | 12.665 | 10.376 |
| *Death* | 1.436 | 1.832 | 27.035 |

*Note: Model performance was assessed using country-specific 5-fold time series cross-validation to ensure temporal ordering of data. Performance metrics represent the mean values across all cross-validation folds, providing an evaluation of model stability and predictive capacity across different national contexts.*


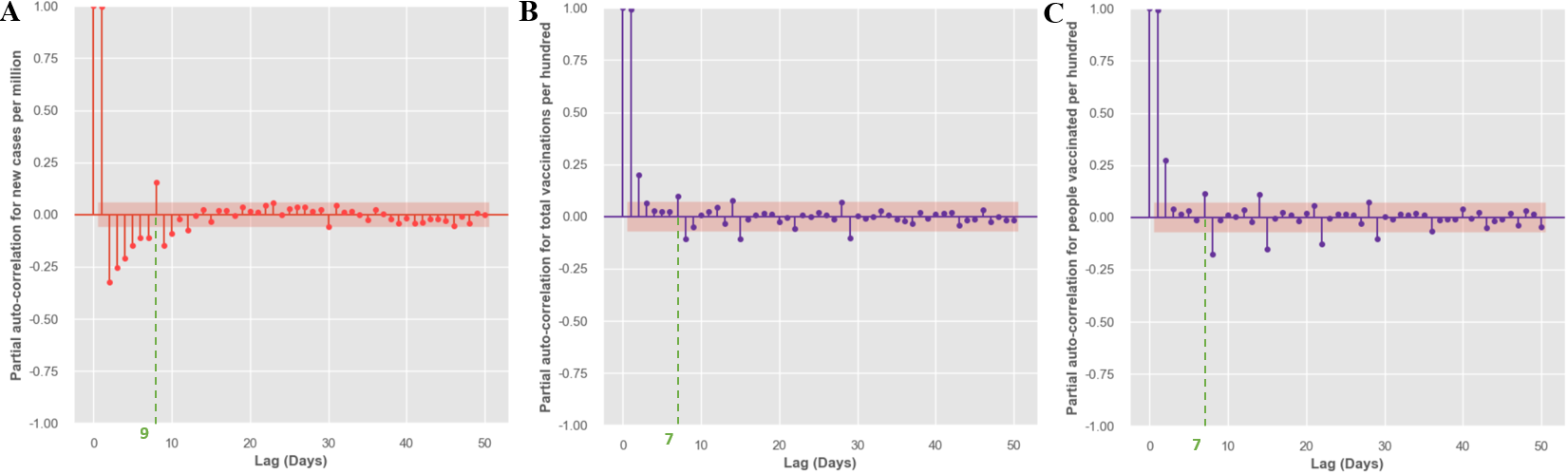


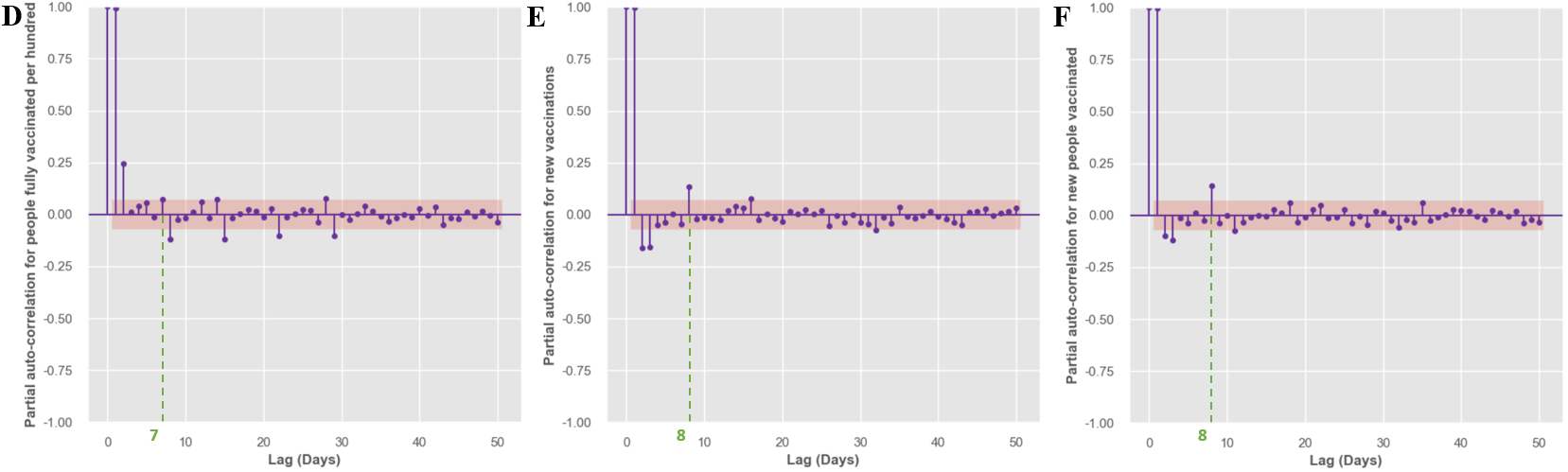


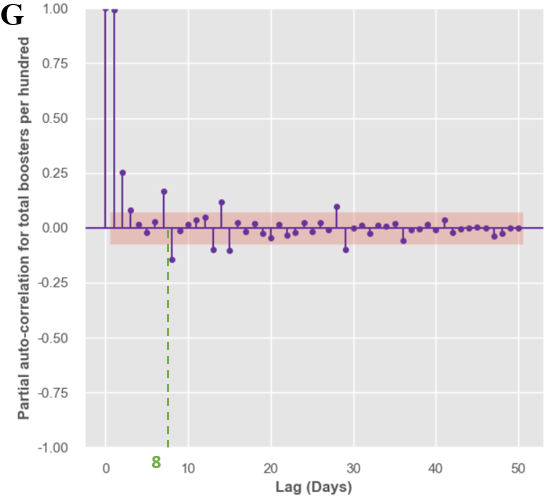


**Figure S1. Determination of lag periods for time-dependent factors influencing COVID-19 metrics.** Partial autocorrelation coefficients were used to identify the optimal lag periods for the following factors: (A) daily new cases per million population, (B) COVID-19 vaccine doses administered per hundred individuals, (C) vaccination rate per hundred individuals, (D) fully vaccinated rate per hundred individuals, (E) daily new vaccine doses administered, (F) daily vaccination rate per hundred individuals, and (G) booster dose coverage per hundred individuals.


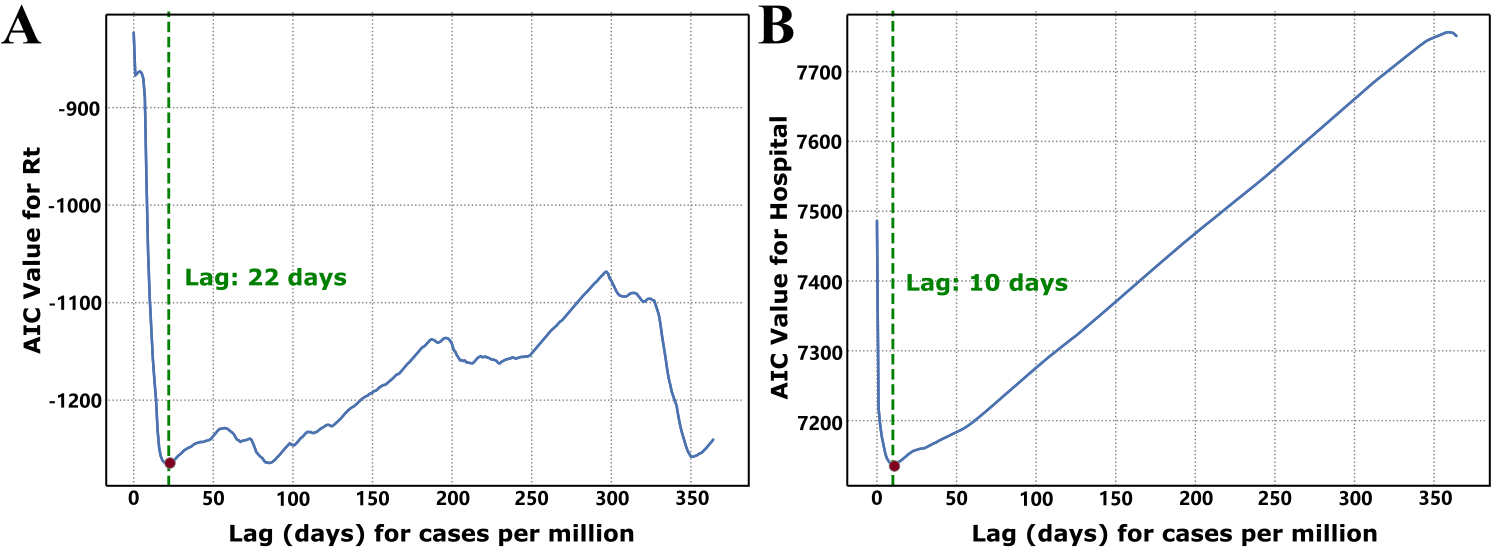


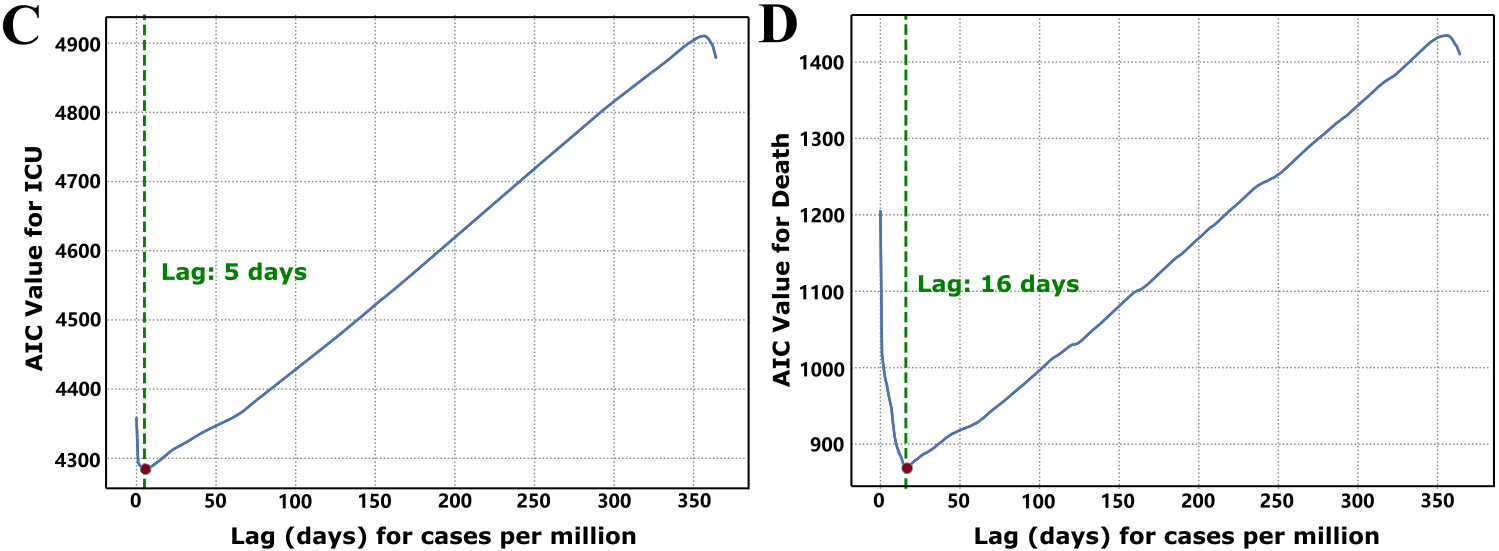


**Figure S2. Identification of optimal lag periods for cumulative infections in influencing COVID-19 burden metrics.** Lagged regression models were utilized to determine the optimal lag periods of total cases per million on the following COVID-19 burden metrics: (A) reproduction number, (B) hospital admissions, (C) critical cases, and (D) mortality burden.


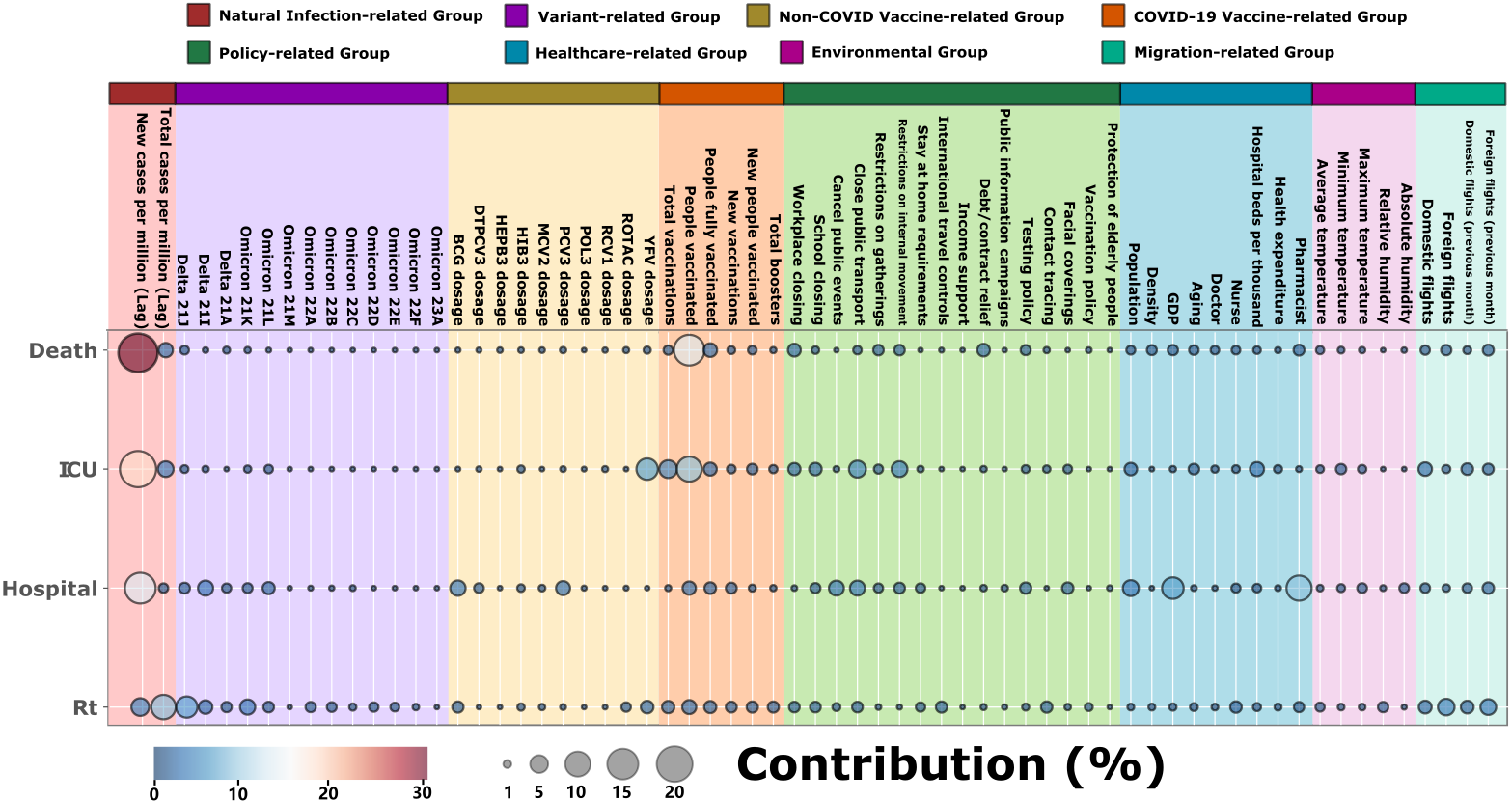


**Figure S3. Contribution of factors to COVID-19 burden metrics**


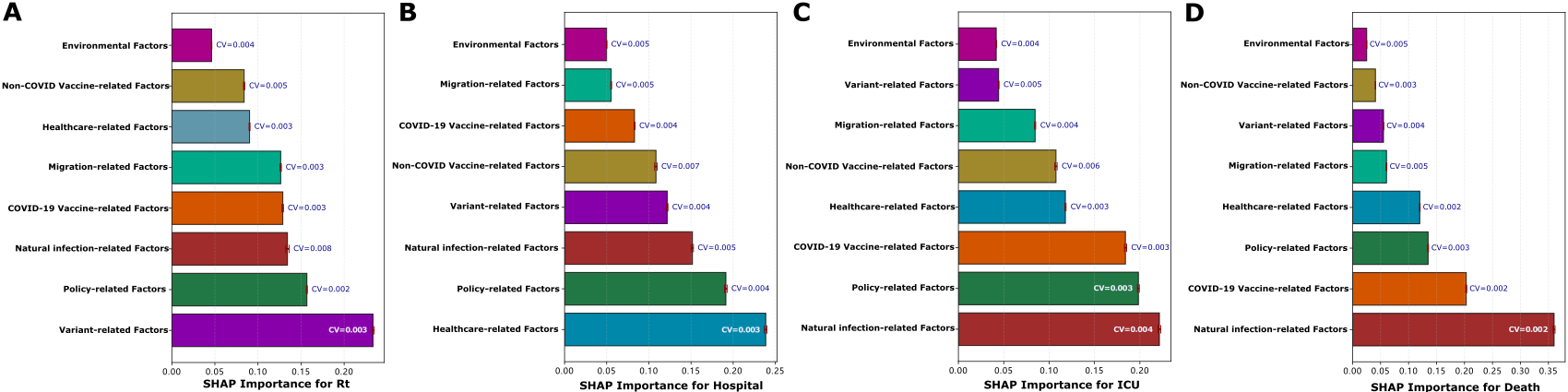


**Figure S4. Bootstrap Stability Analysis of Factor Group Importance Rankings Across COVID-19 Burden Metrics.** Bootstrap validation (n=1000 resamples, 80% sample size) of SHAP-derived factor group importance across COVID-19 burden metrics: (A) Rt, (B) hospitalization, (C) ICU admissions, (D) deaths. Bars show bootstrap mean importance for eight factor groups; error bars represent 95% CIs. Annotated coefficient of variation (CV = SD/mean) quantifies ranking stability: CV < 0.15 indicates high stability, 0.15-0.30 moderate, >0.30 unstable. Bar colors reflect relative importance.


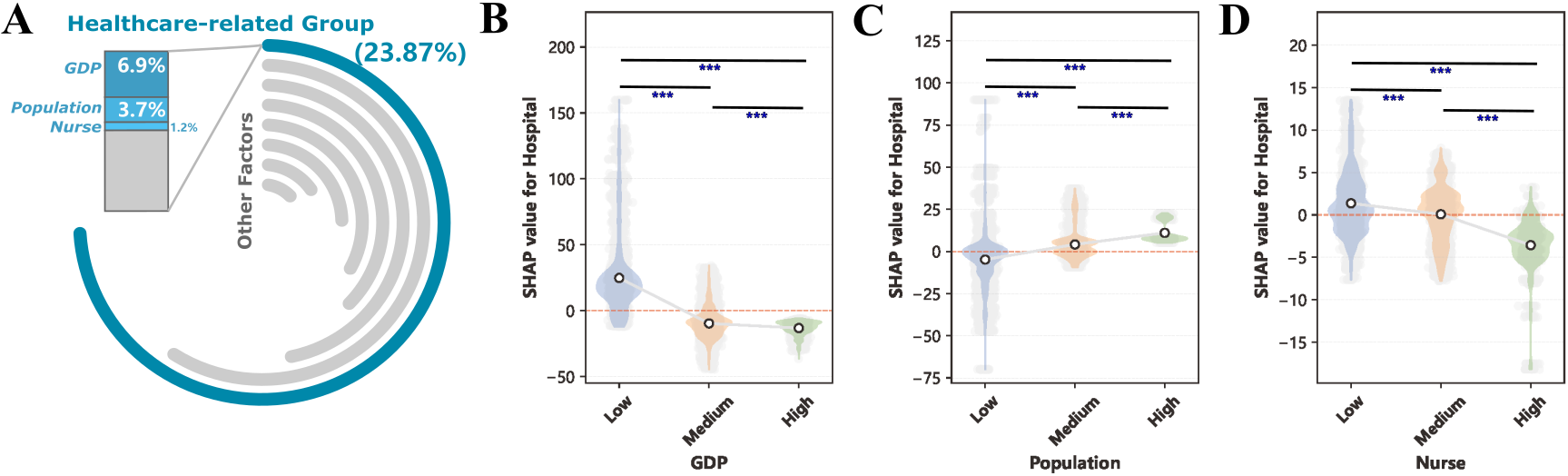


**Figure S5. Impact of Healthcare-Related Group on Hospitalization Burden.** (A) Overall importance of healthcare-related factors on hospitalization burden, highlighting the top three contributors. (B) Effect of GDP per capita on hospitalization burden, categorized into low (<$30,000), medium ($30,000–$60,000), and high (>$60,000) levels. (C) Effect of population size on hospitalization burden, categorized into low (<50 million), medium (50 million–150 million), and high (>150 million) levels. (D) Effect of nurse density (per 1,000 people) on hospitalization burden, categorized into low (<7.5), medium (7.5–15), and high (>15) levels. Differences between categories are statistically significant (*P* < 0.01).


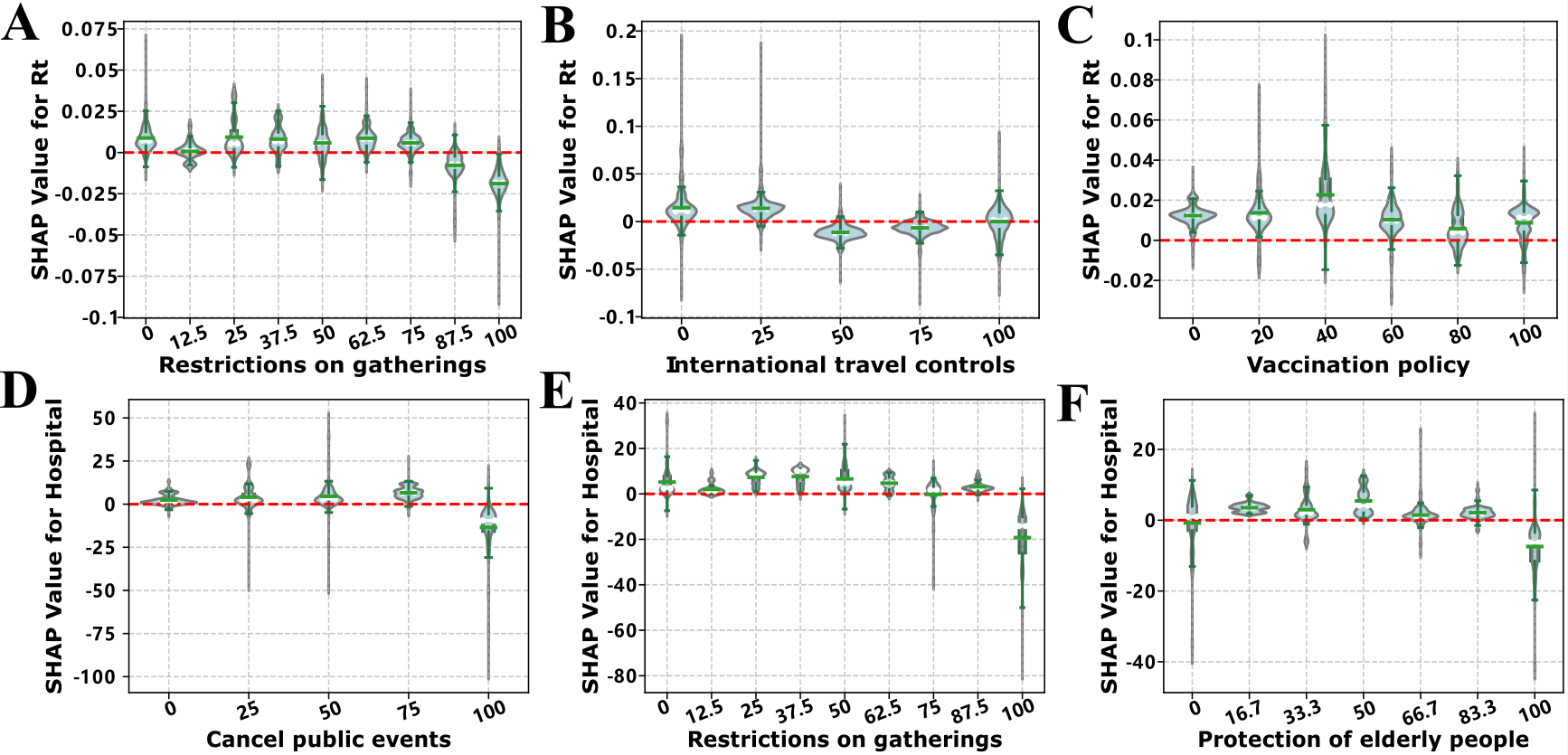


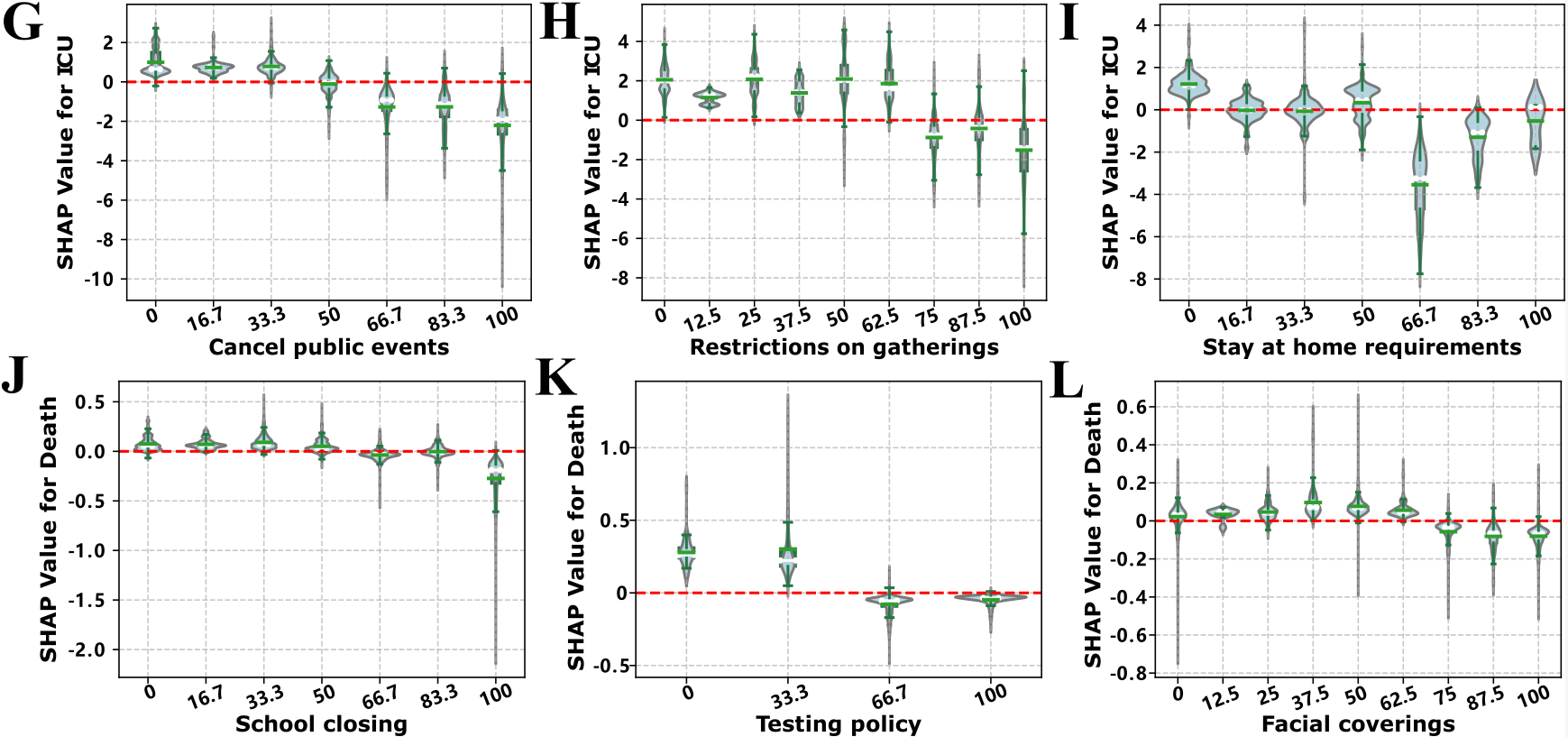


**Figure S6. Impact of the Top Three Influential NPIs on COVID-19 Disease Burden, as measured by their implementation stringency.** The figure illustrates the SHAP values (Y-axis) of NPI stringency (X-axis) on burden metrics: Rt (panels A–C), hospital admissions (panels D–F), ICU admissions (panels G–I), and mortality rates (panels J–L).


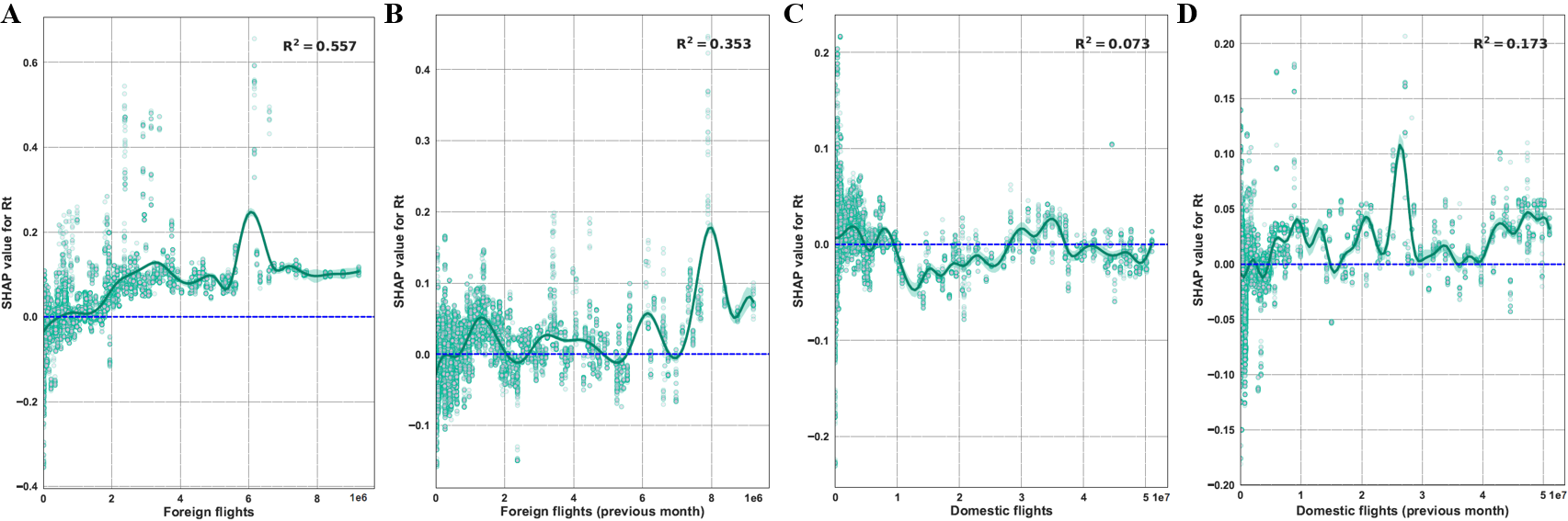


**Figure S7.** **Impact of International, Domestic Air Travel (A, C) and Their Lag Periods (B, D) on the Rt of COVID-19.** The plots depict the nonlinear relationships between migration-related group and Rt based on SHAP values fitted using GAM with 95% confidence intervals (shaded areas).


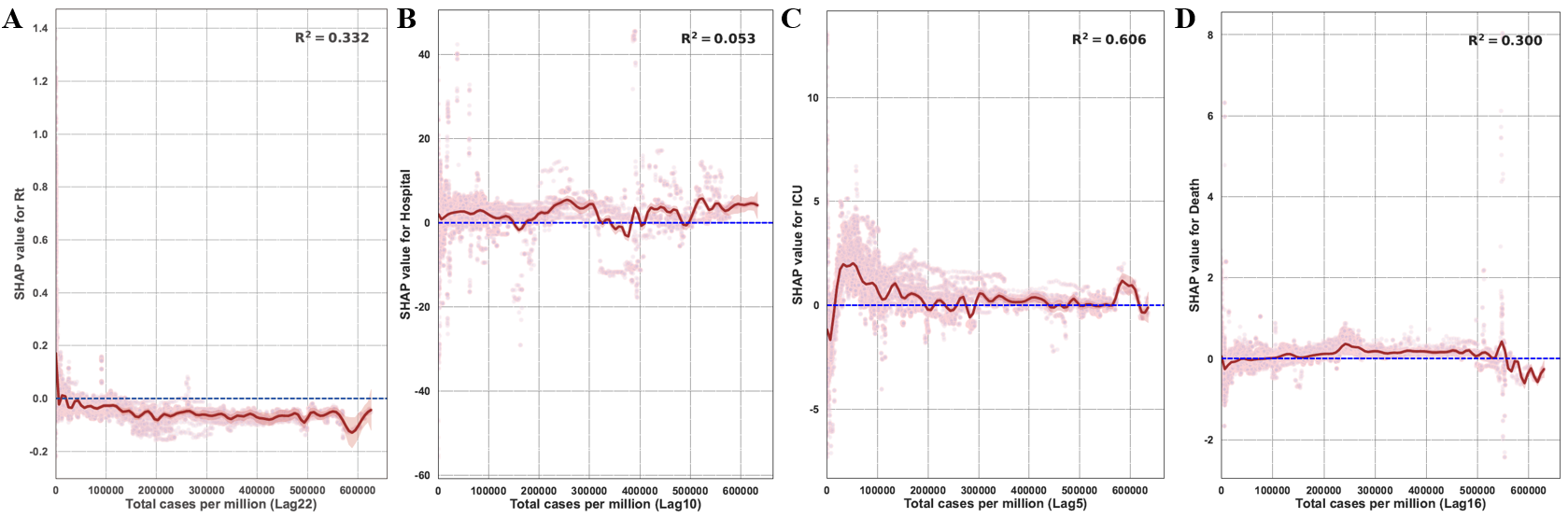


**Figure S8. Nonlinear Effects of Cumulative Natural Infections on COVID-19 Burden Metrics.** The plots depict the nonlinear relationships between natural infections and COVID-19 burden metrics based on SHAP values fitted using GAM with 95% confidence intervals (shaded areas). Panels (A) - (D) illustrate the effects of cumulative natural infections on Rt, hospitalizations, ICU admissions and mortality rates, respectively.


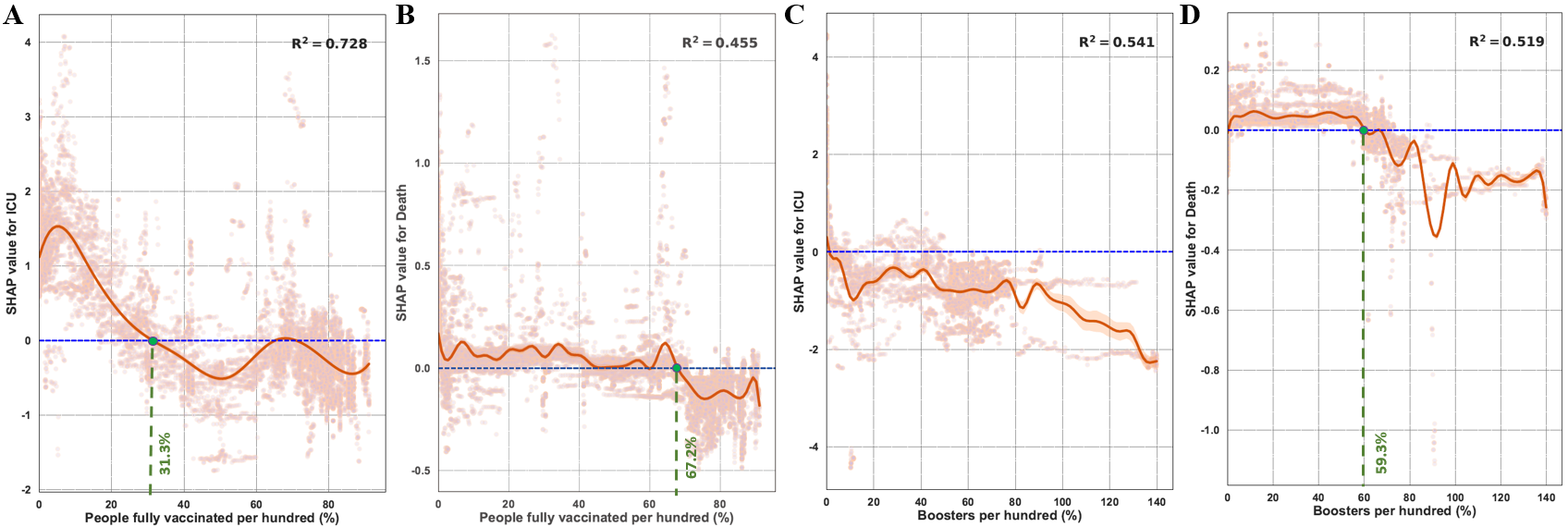


**Figure S9. Nonlinear effects of full vaccination and booster vaccination rates (per hundred, %) on severe COVID-19 burden metrics.** The plots illustrate the nonlinear relationships between vaccination coverage and COVID-19 burden metrics based on SHAP values fitted using GAM with 95% confidence intervals (shaded areas). Panels (A) and (B) show the effects of full COVID-19 vaccination rates on ICU admissions and mortality rates, respectively. Panels (C) and (D) depict the effects of booster vaccination rates on ICU admissions and mortality rates, respectively.


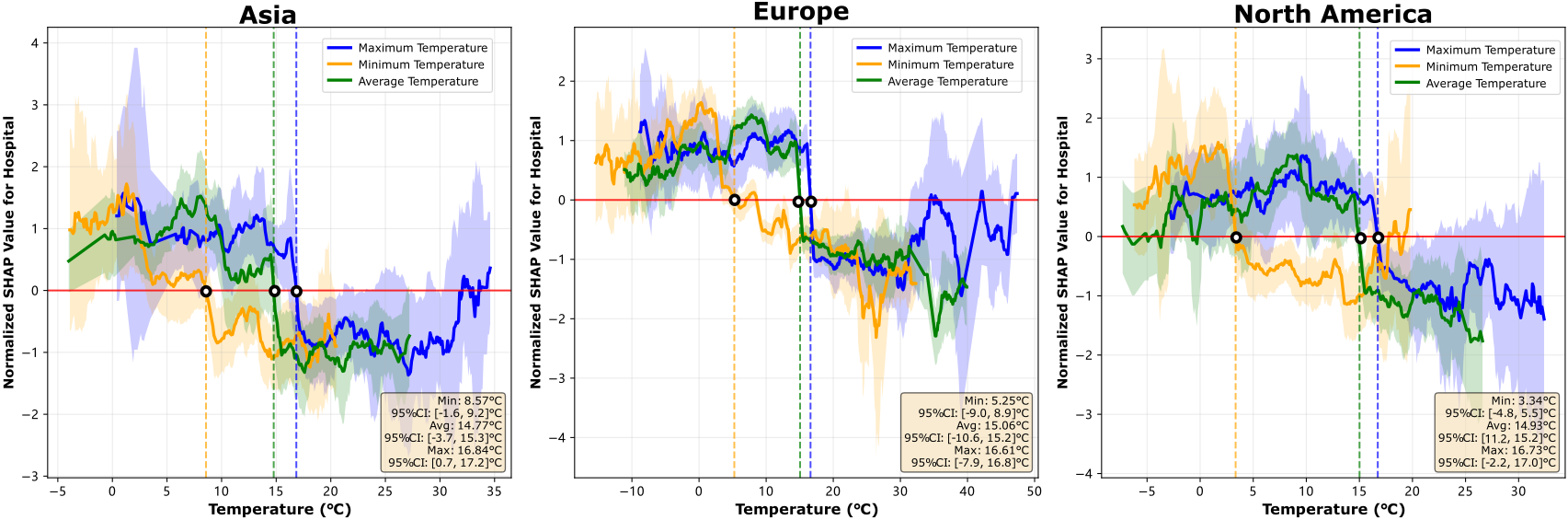


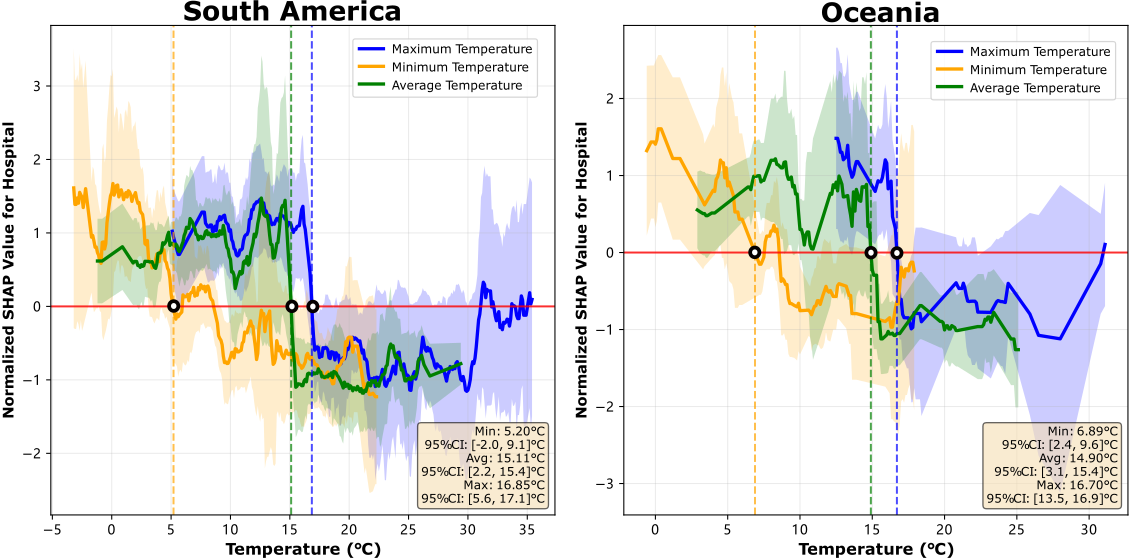


**Figure S10.** **Impact of temperature on COVID-19 hospitalization across continental contexts.** Effects of mean, minimum, and maximum temperature on hospitalization burden across five continents.


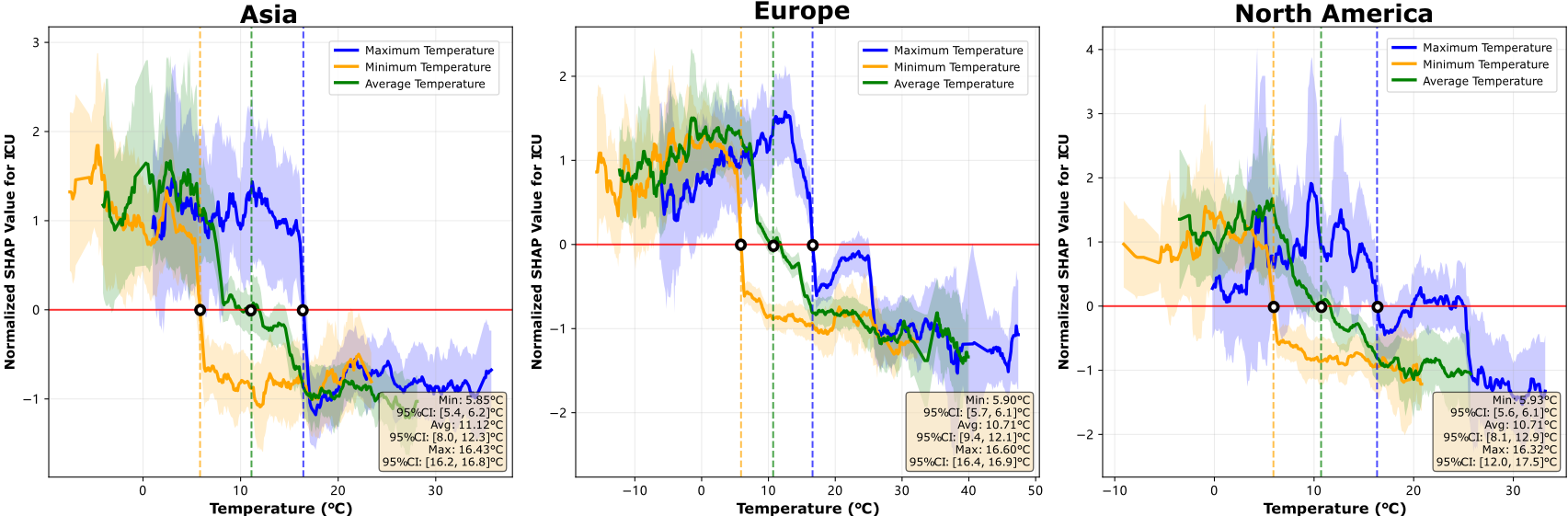


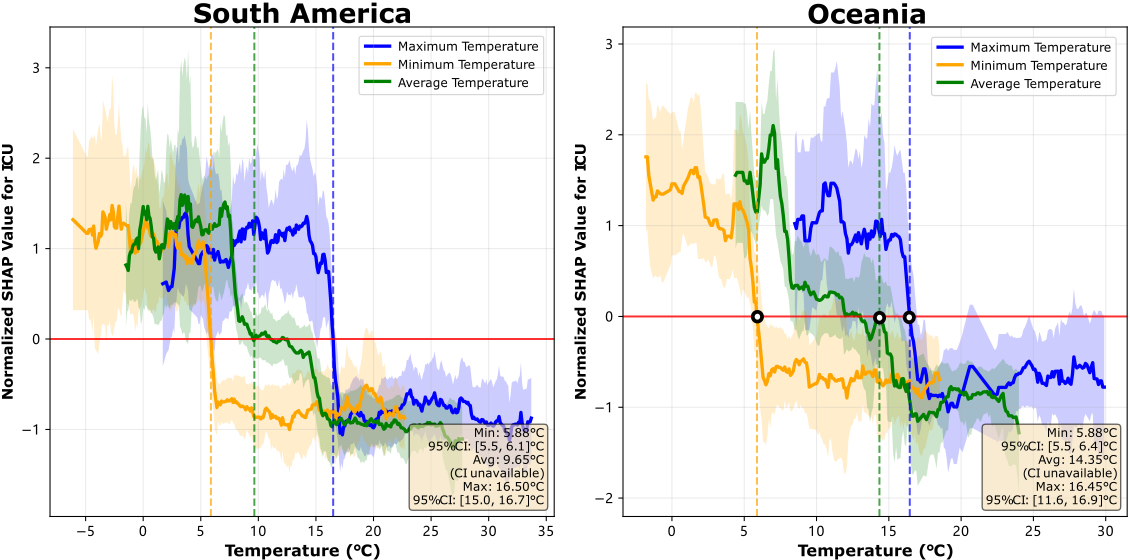


**Figure S11.** **Impact of temperature on COVID-19 ICU admissions across continental contexts.** Effects of mean, minimum, and maximum temperature on ICU burden (daily new intensive care unit admissions per million population) across five continents.


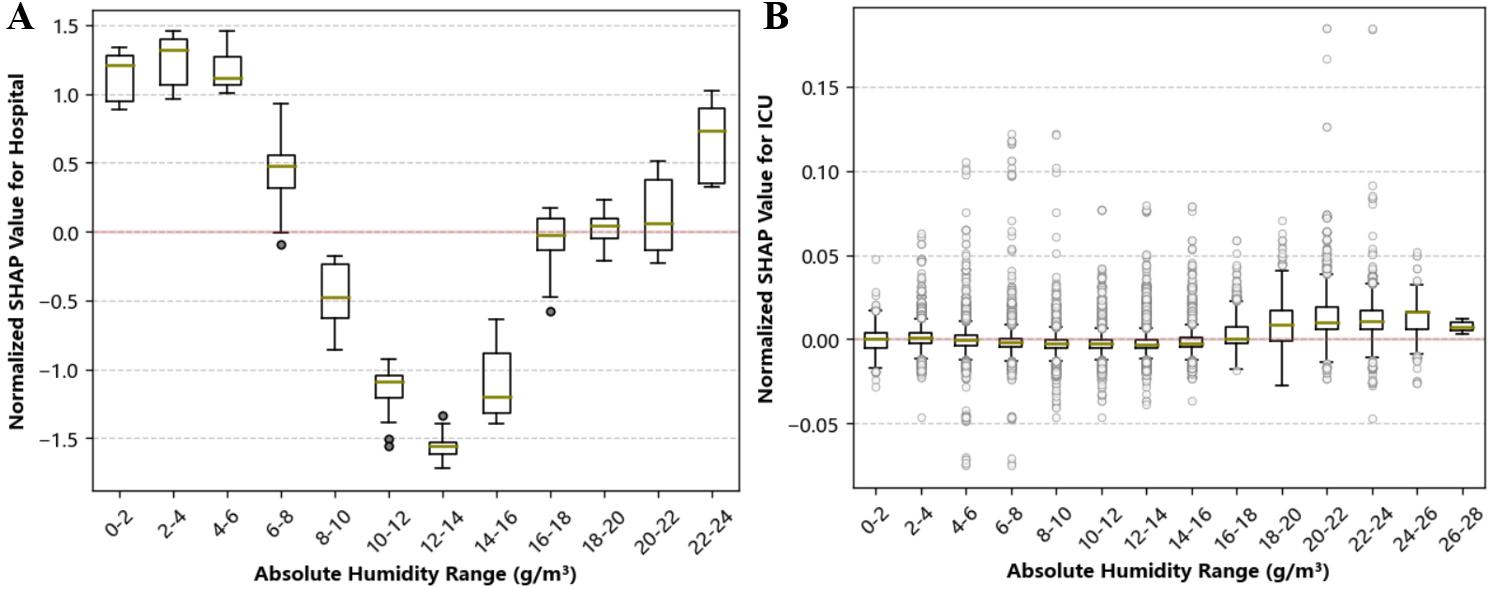


**Figure S12. Effects of Absolute Humidity Range on (A) Hospitalizations and (B) ICU Admissions**


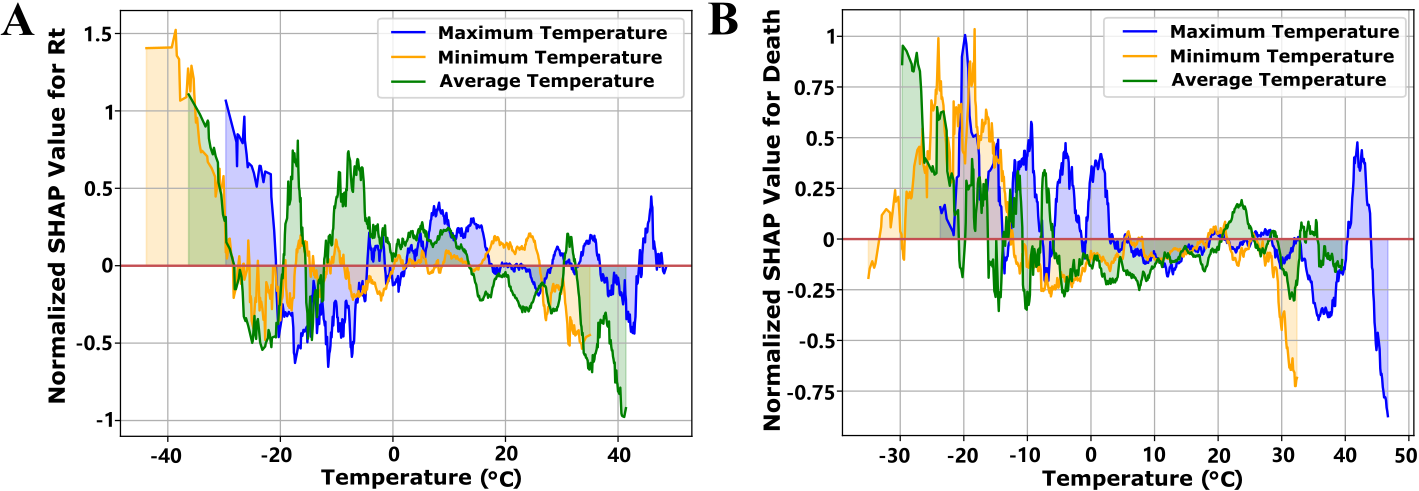


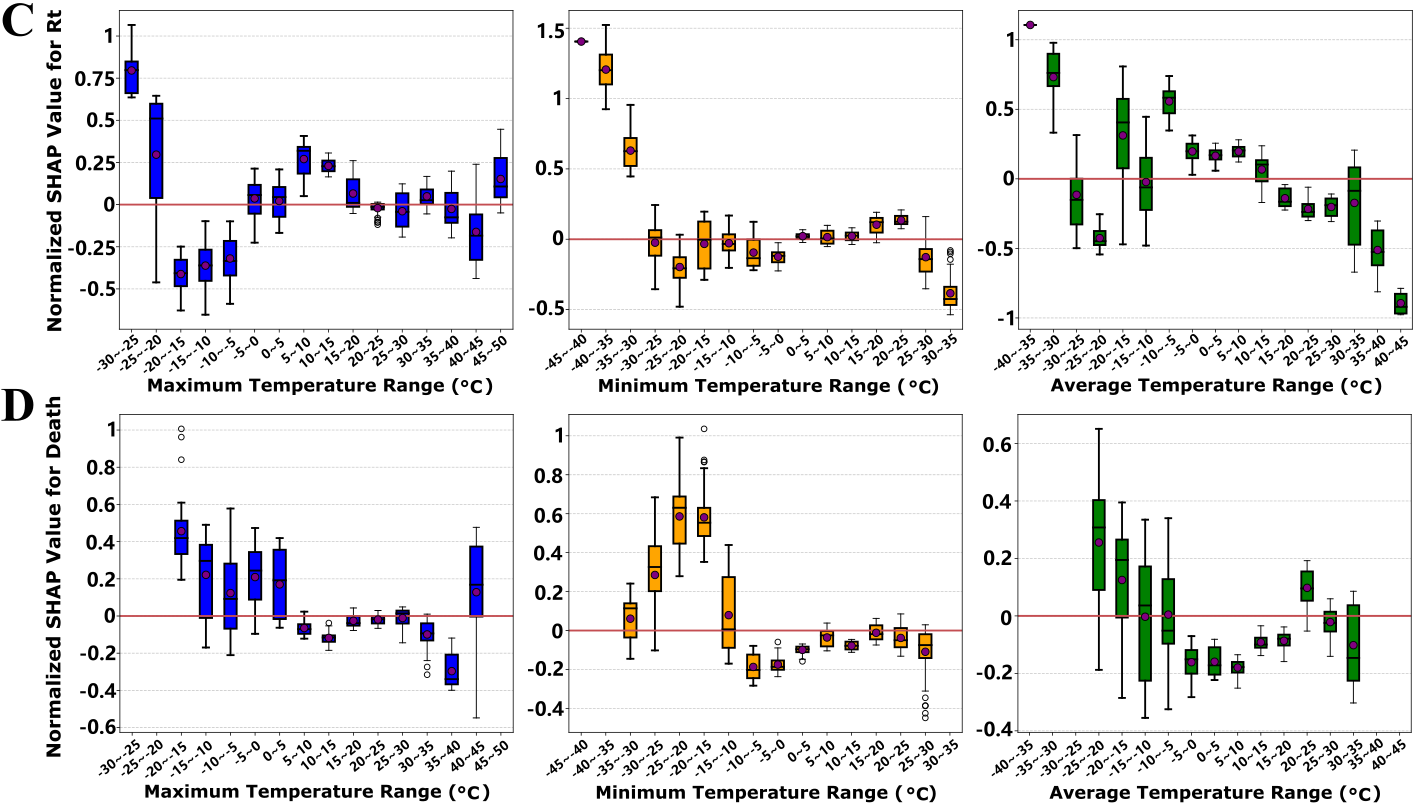


**Figure S13. Dynamic Effects of Temperature Factors on COVID-19.** (A) Association between temperature metrics (mean, maximum, and minimum temperature) and SARS-CoV-2 transmissibility (Rt), expressed as Z-score normalized SHAP values. (B) Temperature-dependent patterns of COVID-19 mortality burden, quantified through standardized SHAP values. (C) Box-and-whisker plots depicting the distribution of temperature-specific effects on Rt across 5°C intervals, with outliers represented as individual points. (D) Temperature interval analysis for mortality outcomes, displaying the heterogeneity of effects across different temperature ranges.


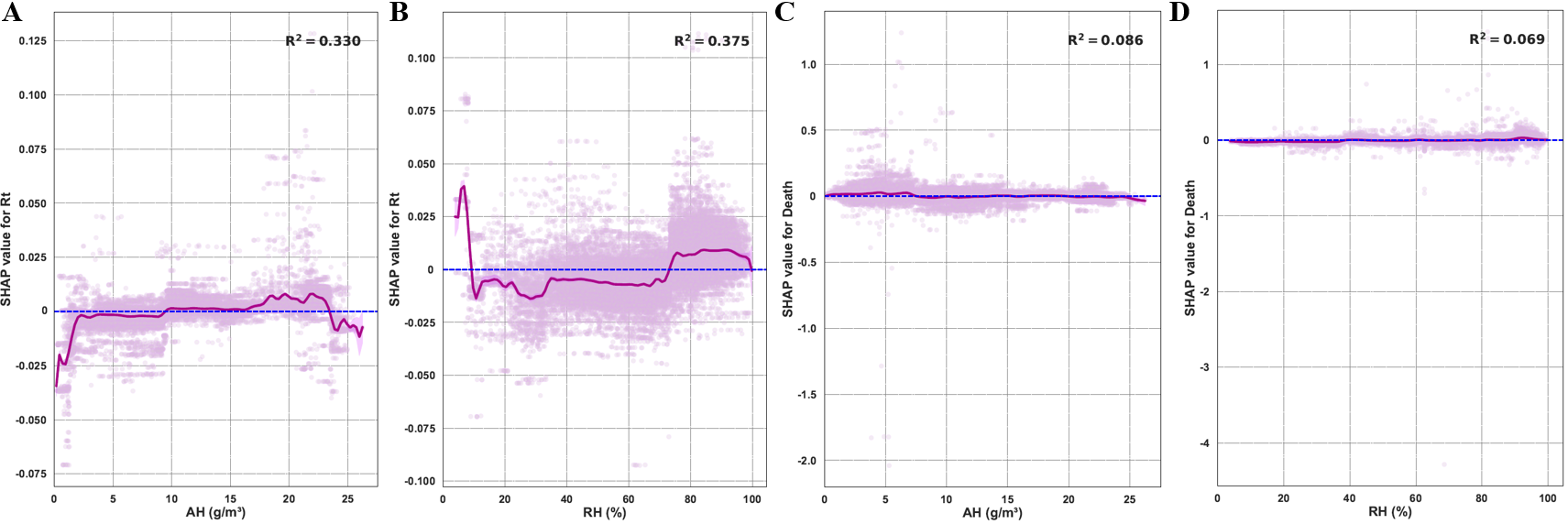


**Figure S14. Dynamic Effects of Humidity Factors on COVID-19.** Humidity-dependent patterns of COVID-19 transmission (A/B) and mortality rates (C/D), showing both absolute humidity (AH) and relative humidity (RH) effects. The relationships were modeled using GAM with smoothing splines, with shaded areas representing 95% confidence intervals of the fitted curves.
